# Quantitative Live Imaging of Human Embryonic Stem Cell Derived Neural Rosettes Reveals Structure-Function Dynamics Coupled to Cortical Development

**DOI:** 10.1371/journal.pcbi.1004453

**Published:** 2015-10-16

**Authors:** Omer Ziv, Assaf Zaritsky, Yakey Yaffe, Naresh Mutukula, Reuven Edri, Yechiel Elkabetz

**Affiliations:** 1 Department of Cell and Developmental Biology, Sackler School of Medicine, Tel Aviv University, Ramat Aviv, Tel Aviv, Israel; 2 Department of Cell Biology, UT Southwestern Medical Center, Dallas, Texas, United States of America; 3 The Blavatnik School of Computer Science, Tel Aviv University, Ramat Aviv, Tel Aviv, Israel; University of California, Berkeley, UNITED STATES

## Abstract

Neural stem cells (NSCs) are progenitor cells for brain development, where cellular spatial composition (*cytoarchitecture*) and dynamics are hypothesized to be linked to critical NSC capabilities. However, understanding cytoarchitectural dynamics of this process has been limited by the difficulty to quantitatively image brain development in vivo. Here, we study NSC dynamics within *Neural Rosettes—*highly organized multicellular structures derived from human pluripotent stem cells. Neural rosettes contain NSCs with strong epithelial polarity and are expected to perform apical-basal interkinetic nuclear migration (INM)—a hallmark of cortical radial glial cell development. We developed a quantitative live imaging framework to characterize INM dynamics within rosettes. We first show that the tendency of cells to follow the INM orientation—a phenomenon we referred to as *radial organization*, is associated with rosette size, presumably via mechanical constraints of the confining structure. Second, early forming rosettes, which are abundant with founder NSCs and correspond to the early proliferative developing cortex, show fast motions and enhanced radial organization. In contrast, later derived rosettes, which are characterized by reduced NSC capacity and elevated numbers of differentiated neurons, and thus correspond to neurogenesis mode in the developing cortex, exhibit slower motions and decreased radial organization. Third, later derived rosettes are characterized by temporal instability in INM measures, in agreement with progressive loss in rosette integrity at later developmental stages. Finally, molecular perturbations of INM by inhibition of ACTIN or NON-MUSCLE MYOSIN-II (NMII) reduced INM measures. Our framework enables quantification of cytoarchitecture NSC dynamics and may have implications in functional molecular studies, drug screening, and iPS cell-based platforms for disease modeling.

## Author Summary

Brain development is a dynamic and complex process that requires highly orchestrated interaction between neural stem cells. Therefore, investigating these dynamics is fundamental for understanding brain development and disease. However, difficulties to record and quantify neural stem cells behavior inside the brain pose a major limitation. We were recently able to mimic brain development in the Petri dish by generating highly organized multicellular structures containing human neural stem cells termed Neural Rosettes. Here we present a newly developed method to record, quantify and analyze the dynamic movements of neural stem cells within rosettes as reflection of their behavior inside the developing brain. We first confirmed that neural stem cells move radially in rosettes similarly to authentic stem cells residing in the developing brain. We then defined novel measures to assess how well these neural stem cells organize into rosettes in culture and found that organization decreases as stem cells progress in culture. Finally, we demonstrated that disruption of rosette structures by specific drugs impairs organization dynamics of neural stem cells. Our findings offer a first insight into neural stem cell dynamics during brain development, and a potential methodology for functional studies and drug discovery.

## Introduction

Neural stem cells (NSCs) are neural progenitor cells within the nervous system that are defined by their ability to self-replicate while retaining potential for generating neurons and glia [1–3]. During nervous system development, early emerging NSCs first undergo successive symmetric cell divisions that generate additional progenitor cells, resulting in expansion of the NSC pool. This phase is then followed by asymmetric cell divisions that generate a progenitor cell and a terminally differentiated cell such as a neuron or a glial cell, resulting in decreased NSC ratios [4] (for review see Ref. [5]). Together, these mechanisms are fundamental for the generation of distinct types of NSCs to account for cellular diversity of the nervous system.

Much progress has been made towards understanding the factors that regulate the self-replication or differentiation of NSCs both in vivo and in vitro [6,7]. One predominantly exciting but less understood aspect of stem cell biology is how the cytoarchitecture of the developing brain is linked to maintenance of NSC number and developmental potential. Particularly interesting is the nuclei dynamics within neuroepithelial cells and radial glial cells—the NSCs that build the cortex. These are elongated cells harboring distant apical and a basal processes that connect the two walls of the developing neural vesicles. Structurally, this layer of NSCs is pseudostratified; i.e., although several layers of nuclei are apparent between vesicle walls, the cytoplasm of each cell extends to contact both apical and basal surfaces of the wall, resulting in a bipolar cellular morphology that is much longer then the thickness of a single cell. Interkinetic nuclear migration (INM) is the process by which nuclei migrate between apical and basal ends of these pseudostratified neuroepithelial cells, in coupling with cell division at the apical surface. INM was first suggested by Sauer [8], further confirmed experimentally [9–11] and later suggested as a mechanism to ensure maintenance of sufficient NSC pools throughout embryonic neocortical development [12]. Thus, it is hypothesized that INM spatial composition and dynamics reflect critical abilities of NSCs during self-renewal and differentiation.

Time-lapse microscopy of mouse and human cortical slices excised from brain and grown in vitro has confirmed INM as a live dynamic process [13] and provided initial clues on mechanisms and functions. Specifically, a correlation between INM and cell cycle was established [14,15] and variations between apical vs. basal INM speed as well as motion patterns were shown in vivo [16]. INM dynamics is fascinating also because the pseudostratification of neuroepithelial cells entails high cellular traffic due to accommodation of many moving nuclei within a limited space. It was suggested that unsynchronized INM may serve as a mechanism to maximize probability of cell division at apical sites and consequent exposure to Notch signaling activation within these sites, which in turn promote maintenance of NSC fate following cell division [12].

Yet, very little is known regarding specifics of INM dynamics, reflecting the technical challenge to image and quantify multiple cells in vivo at high resolution and high content. Therefore, two components are needed in order to advance the field: (1) an in vitro model system that is physiologically relevant and reflects dynamics similar to those observed in vivo, (2) quantitative readouts for cell dynamics in such a system.

Here we used neural rosettes as an in vitro model to explore INM dynamics with relation to NSC maintenance. Neural rosettes are highly organized structures that appear in culture following differentiation of human pluripotent stem cells into cortical lineages. Neural rosettes contain NSCs resembling neuroepithelial and radial glial cells of the developing cortex that are radially organized to create a lumen, resembling the structure of the ventricular zone of the developing cortex. Initial characterization of neural rosettes revealed strong apicobasal cell polarity with the organization of apical ends surrounding a lumen [17]. Joining apical ends at rosette lumens also coincide with mitosis in these luminal regions [17,18], in accordance to INM observations in vivo [19]. We recently dissected the entire cortical differentiation process—from neuroepithelial cells towards distinct radial glial cell types. Specifically, we identified two rosette stages in culture corresponding to two developmentally distinct types of radial glial cells [20]. Early radial glial (E-RG) rosettes (day 14 in culture) contain highly proliferative NSCs exhibiting broad differentiation potential and minimal differentiation propensity in culture [20]. As cultures proceed in vitro, E-RG rosettes progress into a stage termed Mid radial glial (M-RG) rosettes (day 35 in culture), which is characterized by decreased NSC numbers and increased propensity to differentiate into neurons. Further culture of M-RG rosettes (beyond day 55) ultimately results in loss of rosette integrity, a further reduction in NSC numbers, and transition of fate potential from neuronal towards glial bias [20]. We further comprehensively dissected the regulatory networks that drive differentiation from pluripotent stem cells to E-RG and then to M-RG rosettes by employing extensive transcriptional and epigenetic characterization coupled with computational analysis [21]. Together, these findings propose a functional link between cortical development, NSC capacity maintenance and neural rosettes formation and disassembly. Thus, we hypothesized that INM dynamics within neural rosettes may predict NSC capacity of the developing cortex.

Here, we developed quantitative live imaging to characterize cellular dynamics within rosette structures, and investigated these kinetic properties during transition across developmentally distinct rosettes and following molecular perturbations. We devised three main readouts associated with INM to capture different aspects of cell dynamics that relate to migration orientation and speed. We further applied these measures to reveal inherent and stage-dependent observations that distinguish E-RG from M-RG rosettes. Finally, we were also able to detect and quantify reduction in cellular organized dynamic performance following specific inhibition of molecular motors involved in INM. Thus, our quantitative approach delineates a model that describes intrinsic dynamic features within rosettes and suggests for the first time a functional link between rosette dynamics and NSC competence. Hence, the presented kinetic quantitative readouts have the potential to serve for functional molecular studies drug screening, and may be implicated to gain novel insights into biology of NSCs in health and disease.

## Results

### Quantifying radial patterns of cell dynamics in neural rosettes

**Fig 1:**
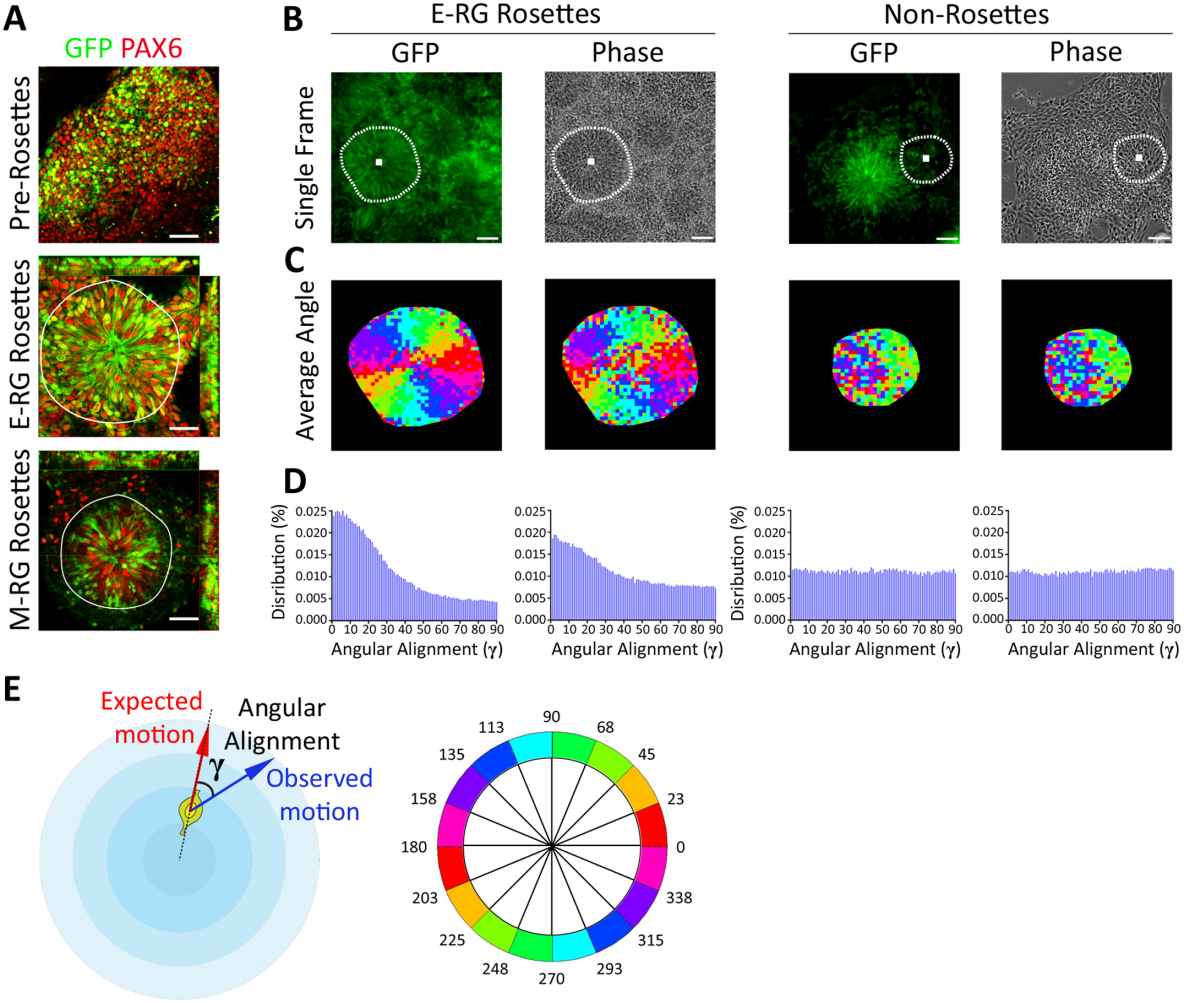
Radial patterns of cell dynamics in neural rosettes. **A.** Combined *HES5*::*eGFP* (green) reporter expression and immunostaining of the cortical neural progenitor marker PAX6 (red) throughout NSC progression from unstructured neuroepithelial cells (top), to early radial glial (E-RG) rosettes (middle) to mid radial glial (M-RG) rosettes (bottom). Nuclei are stained with DAPI (blue). Rosette contours are marked in white. Scale bars: 25 μm. HES5::eGFP co-localizes with PAX6+ nuclei, attesting a NSC stage. E-RG rosettes contain multiple radially organized GFP+/PAX6+ nuclei, whereas M-RG rosettes harbor GFP+/PAX6+ cells only close to rosette lumens, reflective of enhanced or reduced NSC numbers, respectively. Many cells in M-RG rosettes are not associated to apical sites (e.g., rosette lumens), reflecting the beginning of rosette disassembly. **B.** Representative *HES5*::*eGFP* and its matched phase contrast image from time-lapse imaging of an E-RG stage neural rosette (left panels) or a non-rosette area adjacent to a rosette (right panels). Rosette contours and center were manually annotated (white dashed marking). Scale bars: 25 μm. An image was acquired every 5 minutes for a total of 250 minutes. Rosette annotation for M-RG rosettes is shown in S1A Fig. **C.** Motion patterns follow the expected radial angle. Average patch velocity orientation over time for an E-RG rosette (left, corresponding to panel B) and a non-rosette. Color code is illustrated in panel E (bottom). Radial organization is subjectively observed in E-RG rosettes, for both GFP and phase contrast, but not in non-rosettes. **D.** Distributions of angular alignment of all patches over the entire time course. INM patterns (tendency to follow the expected angle) are found for E-RG rosettes (left, mean angle of 29° for GFP, 36.4° for phase contrast) but not for non-rosettes (right, mean angle of 44.7° for GFP, 45.7° for phase contrast). **E.** Schematic sketch of angular alignment **γ**, the angle between the expected- and observed-motion (top). Color code for angles is illustrated in panel C (bottom).

We used the *HES5*::*eGFP* Notch activation reporter human embryonic stem cell (hESC) line, expressing cytoplasmic GFP in Notch active cells [22]. HES5 is a major and direct downstream target of Notch activation pathway (for review, see Ref. [23]) and specifically marks NSCs in vivo. We recently showed that neural rosettes correspond to NSCs of the developing cortex based on their strong apico-basal epithelial polarity and the expression of cortex associated genes such as PAX6 together with the NSC marker *HES5*::*eGFP* [20] (Fig 1A). When newly formed E-RG rosettes appear in culture on day 14, most rosette cells express the cortical marker PAX6 and the NSC marker *HES5*::*eGFP* (Fig 1A, middle panel) (>80%; see Ref. [20]) in accordance with their high proliferative capacity and lack of differentiation in culture. In contrast, continued culture of E-RG rosettes results in their progression towards M-RG rosettes around day 35, and this is marked by significant loss in the NSC marker *HES5*::*eGFP* and the cortical marker PAX6 in rosette cells (Fig 1A, bottom panel) (<30%; see Ref. [20]). Importantly, PAX6 expression is now limited only to the regions adjacent to rosette lumens, reflecting the limited area where stem cells reside at that stage. Since the ability of neural progenitors to radially organize in rosettes is correlated with increased ratios of polarized epithelial NSCs [17,18,20], we hypothesized that this difference in NSC numbers among early and advanced rosettes would be phenotypically reflected in rosette dynamics, specifically that of INM.

Subjective live imaging observations suggested that both E-RG and M-RG rosettes exhibit INM characteristics, and this was even apparent in matching phase contrast images (S1 and S3 Movies; compare to non-rosettes, S2 Movie; phase contrast time lapses immediately follow GFP time lapses in each movie). To quantitatively validate the observed INM motility-patterns, we devised an automated objective framework to assess rosettes dynamics. The analysis was based on manual annotation of rosette contours and centers from the phase-contrast channel, where the cytoarchitecture outlines of the region performing INM become obvious to a human eye based on different texture-patterns in the image (Figs 1B, right panels and S1A; See Methods). Rosette outlines remained stable in the culture dish and did not change throughout the experiment (S1B Fig). Rosette areas were discretized to sub-cellular *patches* (S2A Fig; See Methods) and local cross-correlation was applied to estimate motion for each patch at each time point [24], similarly to particle image velocimetry (PIV) [25,26]. This approach was validated as highly correlative to manual single-cell tracking (S2B Fig). Motions were exceptionally fast ranging up to 120μm hr^-1^ (S2C Fig), and only motions of 15μm hr^-1^ or faster (≥ 2 pixels per time-lapse frame) were considered for further analysis.

We first estimated the average velocity orientation for each of the coordinates within each rosette over the entire time course (Fig 1C, left panels). Indeed, migration pattern followed the *expected radial angle*, as defined by orientation of each patch’s velocity relatively to the rosette center, i.e., the expected velocity direction assuming apico-basal radial motion (See schematics in Fig 1E). This pattern was also reflected by the normal distributions observed for motion angles grouped by their expected radial angle (S2D Fig). As expected, this pattern did not occur for cells in non-rosette areas that were adjacent to rosettes (Fig 1C, right panels).

More quantitatively, we calculated the distribution of the *angular alignment* γ between the observed velocities and their respective expected radial angles (See schematics in Fig 1E) to validate a dramatic bias of the angular alignment distribution toward structured motion (Fig 1D, compare E-RG Rosettes, left, to Non-rosettes, right). Strikingly, this dynamics was even obvious also when computing motions based on the phase contrast channel, further confirming that rosettes indeed perform radial migration (Fig 1B–1D, left, compare GFP columns to Phase columns). These observations indicate that motion within rosettes resemble in vivo INM [8,27] and suggest that radial migration within rosettes in vitro plays a functional role in the maintenance of NSCs.

### Quantitative measures for rosette dynamics

**Fig 2:**
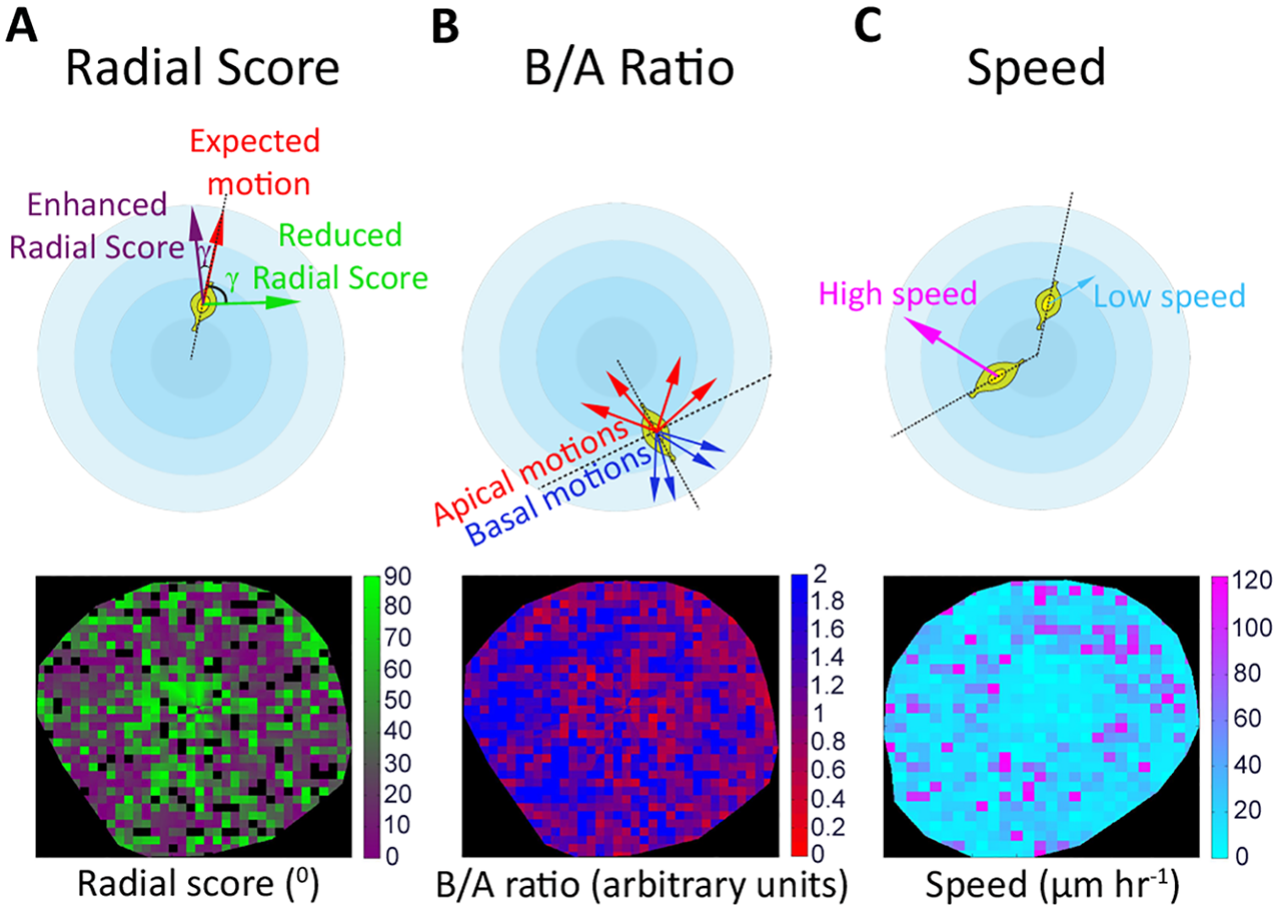
Quantitative measures for rosette dynamics. Schematic sketch (top, circular structure represent a neural rosette) and patches visualization (bottom) for radial score **(A)**, basal-to-apical ratio **(B)** and speed **(C)**, depicted for a representative E-RG rosette. Each patch is assigned its average value over time.

Based on our initial observations we devised three objective measures to study cell dynamics in rosettes to enable functional quantification. Each measure was defined as a scalar readout per rosette that quantifies different aspects in its dynamics throughout time. The first measure, *Radial Score* (RS), was defined as the average angular alignment (γ) of all motions in each rosette over the entire time course (Fig 2A). RS quantifies the mean alignment between observed and expected radial angles. Thus, lower scores correspond to better alignment, reflecting a more organized radial migration (denoted *radial organization* henceforth). The second measure, *Basal to Apical ratio* (B/A ratio), was defined as the ratio between the number of basal (distal) motions to apical (luminal) motions within rosettes along the entire time course (Fig 2B). RS and B/A ratio were designed to quantify INM in vitro, which corresponds to the basal to apical migration observed for the developing neuroepithelium in vivo [16,19]. The third measure, *Speed* was defined as the average magnitude of velocity for all patches across time (Fig 2C), a measure that was quantified in vivo [16,19]. When calculated for each time frame over time, these three measures fluctuated around a mean value, validating that the progressive rosette-disassembly in culture is much slower than the four hour imaging course (S2E Fig), thus allowing us to focus on the mean measures as our readouts.

Based on our observations (Fig 1), we hypothesized that these quantitative measures could reveal inherent biophysical properties of neural rosettes and their associations. These measures may also enable to functionally distinguish between INM performance capacity of E-RG and M-RG rosettes, and further test the hypothesis that the INM of M-RG rosettes is compromised and reflects the beginning of rosette disassembly, in line with the increase in cells with non-epithelial character [17,18,20]. 25 E-RG rosettes and 14 M-RG rosettes were imaged and quantified to test our hypotheses as detailed next.

### Radial organization is associated with rosette size and declines during rosette progression in culture

**Fig 3:**
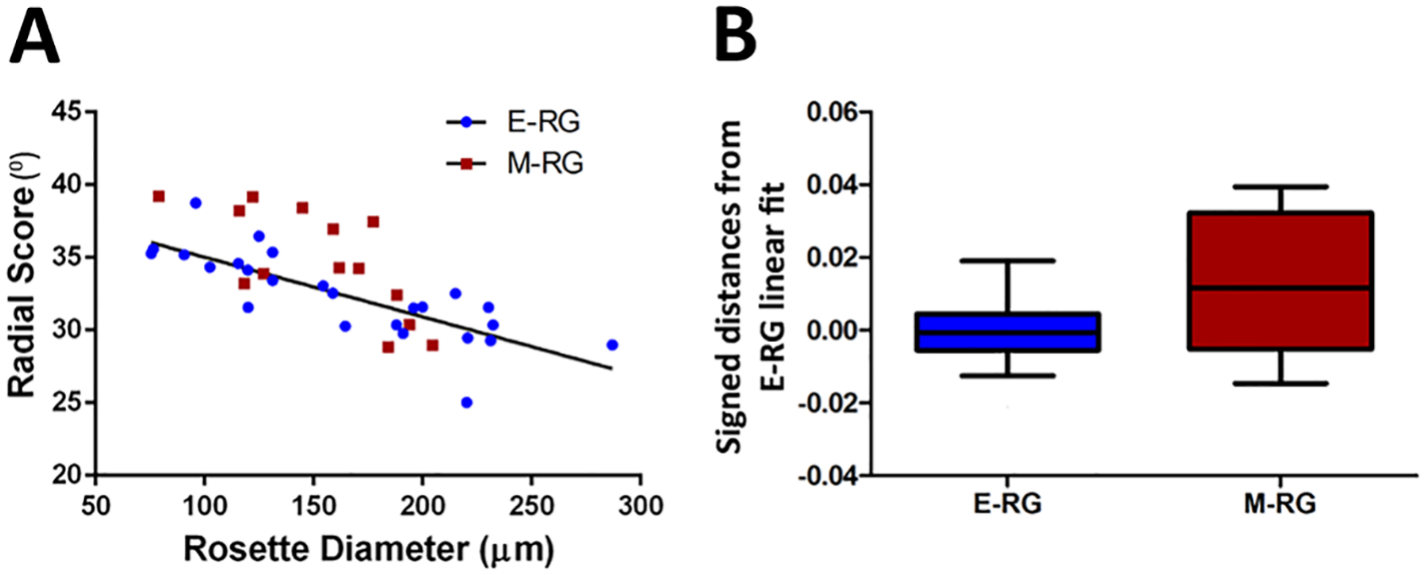
Radial score is associated with rosette size, and enhanced for E-RG rosettes. **A.** Radial score is associated with rosette size. RS of E-RG (Pearson Rho = -0.8, p = 1.55E-06) and M-RG (Pearson Rho = -0.68, p = 0.0112) are associated with rosette size. RS of most M-RG rosettes are above the linear fit of E-RG RS and rosette size (black line), implying reduced radial organization. **B.** Radial score is elevated for E-RG rosettes. Boxplots showing signed distances between RS of E-RG and M-RG rosettes to the linear fit of E-RG RS and rosette size. Value of 0 implies perfect fit, positive values indicate reduced radial organization. E-RG rosettes are characterized by enhanced radial organization (reduced RS) than M-RG rosettes (Wilcoxon rank-sum test, p = 0.048). 25 E-RG rosettes and 14 M-RG rosettes were analyzed.

We hypothesized that larger rosettes are characterized by enhanced structured motion, as a response to increased mechanical constraints by the rosette cytoarchitecture. To test this, we examined the association between RS and rosette size. This property was first examined for E-RG rosettes, speculated to have a more structured dynamics compared to the more advanced M-RG rosettes. Indeed, RS of E-RG rosettes was found to be associated with rosette size (Fig 3A), indicating that larger rosettes exhibit enhanced radial migration. Similarly to E-RG rosettes, RS of M-RG rosettes was also found to be associated to rosette size (Fig 3A), suggesting that the association between rosette size and RS is an intrinsic property. This result also implies that the robustness of INM is augmented when larger numbers of nuclei move together towards apical or basal sites, as previously suggested for INM in pseudostratified neuroepithelial cells in vivo [12]. A similar model works also in other studies of collective cell migration showing enhanced group coordinated motility correlated with group size [28–32]. We therefore suggest that the confined structures of larger rosettes lead to more mechanical constraints that explain the increased radial organization of larger rosettes.

Next, we tested whether M-RG rosettes differ in their radial migration dynamics compared to E-RG rosettes. We found that RS of M-RG rosettes were larger (less organized) than the expected size-dependent values derived from the E-RG’s linear model (Fig 3A, most M-RG rosettes above the line and Fig 3B, quantitatively). We conclude that rosette size plays a prominent role with linear effect on radial migration and that RS can serve as a functional predictive measure for NSC capacity within rosettes.

### Enhanced organization of basal motion contributes to elevated organization of E-RG rosettes

**Fig 4:**
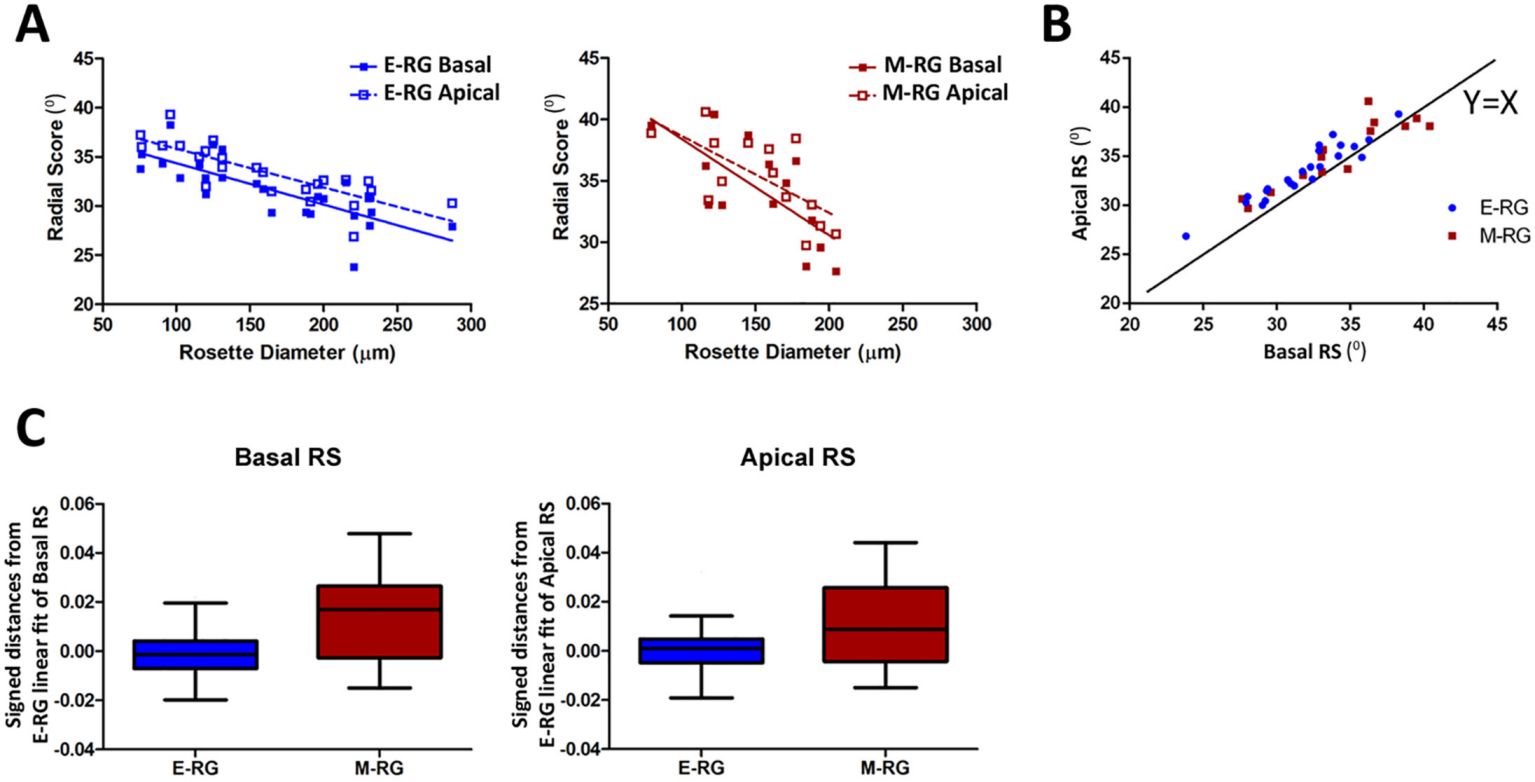
Enhanced basal radial organization contributes to a general elevation in radial organization of E-RG rosettes. **A.** Basal and apical RS are associated with rosette size. E-RG rosettes (Left, Pearson: apical Rho = -0.81, p = 5.77E-07; basal Rho = -0.77, p = 5.61E-06). M-RG rosettes (Right, Pearson: apical Rho = -0.67, p = 0.0088; basal Rho = -0.70, p = 0.0048). Linear fit: dashed line for apical, solid for basal motion. **B.** RS of basal and apical motion are associated (E-RG: Pearson Rho = 0.947, p = 7.883E-13; M-RG: Pearson Rho = 0.899, p = 1.238E-05). Black line y = x, values above this line reflect reduced radial organization (increased RS) of apical motions. **C.** Left, RS of basal motions in M-RG rosettes were significantly increased (reduced radial organization) compared to E-RG rosettes. Boxplots showing signed distances between RS of basal motion in E-RG and M-RG rosettes to the linear fit between RS of basal motions for E-RG rosettes (Wilcoxon rank sum test, p = 0.039). Right, apical RS values in M-RG rosettes were not found to be significantly farther from ERG’s apical score linear model (Wilcoxon rank sum test, p = 0.1173).

Previous in vivo studies have shown differences in speed between nuclei migrating apically and basally [16,33]. We hypothesized that radial organization may also differ between apical and basal motion. To test this hypothesis we classified each motion vector as moving apically (inward) or basally (outward) with respect to rosette center, reflective of apical and basal nuclei migration during INM, and examined apical and basal motion independently. We partitioned the motion vectors of each rosette into basal and apical groups and calculated each group’s RS. We found that similarly to general RS, basal or apical RS were associated with rosette size (Fig 4A). However, basal motion tends to be more radially organized (i.e., lower RS) than apical motion (Fig 4B, most points above the y = x line), regardless of rosette stage (Fig 4B) or size (Fig 4A, basal RS tends to be lower than apical RS for all rosette sizes). This was also judged by the basal RS values for M-RG rosettes, which were higher (less organized) than the basal RS values predicted by the linear fit of E-RG rosettes (Fig 4C). These results led to the hypothesis that enhanced radial organization of basal motions contributes to the overall elevated radial organization observed for E-RG rosettes.

B/A ratio was hypothesized as a secondary mechanism for the elevated radial organization of E-RG rosettes, by enhancing the contribution of the more radially organized basal motions in a size-independent mechanism (S3 Fig, S1 Note).

### Cells in E-RG rosettes exhibit faster motions than M-RG rosettes

**Fig 5:**
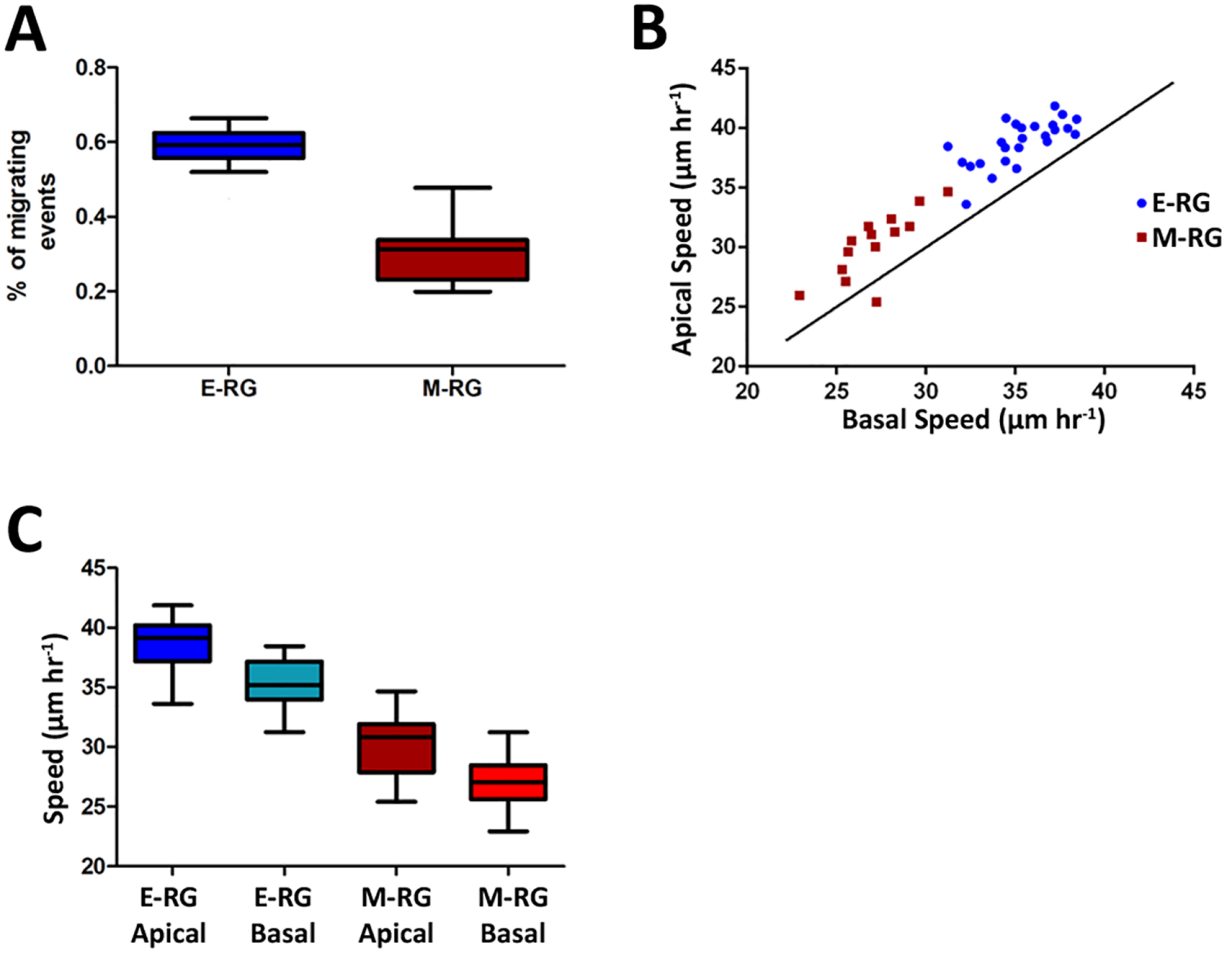
Cells in E-RG rosettes exhibit faster motions than M-RG rosettes. **A.** Two fold increase in percent of highly motile cells (above speed of 15 μm hr^-1^) in E-RG rosettes (average = 0.6) compared to M-RG rosettes (average = 0.3, Wilcoxon rank sum test = 3.7831E-07). **B.** Apical motion was consistently faster than basal motion (Wilcoxon sign rank, E-RG: 1.1 fold, p = 1.229E-05; M-RG: 1.11 fold, p = 3.6621E-04), with higher speed for E-RG rosettes (Wilcoxon rank sum test, apical: 1.28 fold, p = 4.4116E-07; basal: 1.3 fold, p = 3.2415E-07; general speed: p = 3.783E-07). Black y = x line is given as reference. **C.** Apical speed of E-RG rosettes (mean = 38.81μm hour^-1^) > basal speed of E-RG rosettes (mean = 35.27μm hour^-1^) > apical speed of M-RG rosettes (mean = 30.25μm hour^-1^) > basal speed of M-RG rosettes (mean = 27.12μm hour^-1^). 25 E-RG rosettes and 14 M-RG rosettes were analyzed.

The measures RS and B/A ratio were calculated based on the orientation of the velocity vectors. Next we considered the *speed*—the magnitude of these vectors—as a third measure for rosette dynamics (See Fig 2). We found a two fold increase in the fraction of cells moving at speed of 15 μm hr^-1^ or faster in E-RG rosettes compared to M-RG rosettes (Fig 5A). This strikingly fits our recent findings according to which there are twice as much NSCs (i.e., GFP+ cells) in E-RG rosettes compared to M-RG rosettes [20], further drawing a correlation between the actual number of NSCs measured within rosette cultures and the computed quantification of their INM motions. In contrast to RS, rosette speed was not correlated to rosette size (S4 Fig), suggesting that molecular motors driving nuclei motions are less affected by the rosette confining structure. When considering apical and basal motions independently, we found that apical motion was consistently faster than basal motion in a striking linear relation, and with higher speed for E-RG rosettes (Fig 5B). Higher apical (vs. basal) nuclear migration speeds were previously reported in time-lapse ex vivo cultured embryonic cortical slices [16] as well as in vivo in zebrafish retina and brain [33], providing further validation to our quantitative approach as an in vitro platform for investigating INM. Direct comparison further revealed an ordered relation for rosette speed as follows: apical speed of E-RG rosettes > basal speed of E-RG rosettes > apical speed of M-RG rosettes > basal speed of M-RG rosettes (Fig 5C). Inclusively, these data suggest that during early human cortical development, high NSC numbers are accompanied by elevated INM speed towards apical sites, similar to as shown for radial glial cells in cultured cortical slices ex vivo [16].

Our observations indicate that rosettes are generally characterized by basal motions that are slower but more radially organized, while apical motions are relatively faster yet less organized. Also, E-RG rosettes display elevated radial organization and higher speeds in general, for both basal and apical motions, compared to M-RG rosettes (Fig 5B and 5C). These two observations (fast & less organized versus fast & more organized) seem, at first, conflicting, but they are reconciled by the notion that E-RG rosettes exhibit high performance for both basal and apical motions to be faster (Fig 5C) as well as more organized (Fig 3B) than M-RG rosettes. This is while keeping in proportion the inherent hierarchy of basal motions that are generally slower and more organized, compared to apical motions, which are generally faster and less organized, in a rosette stage independent manner. Altogether, these findings also suggest that more than a single mechanism is involved in linking speed to radial organization of INM.

### Spatial dynamics of neural rosettes

We noticed that all measures follow a spatial pattern. It is apparent that inner cells at the center of a rosette exhibit reduced organization, low B/A ratios and slower motion than cells located elsewhere (Fig 2). To understand the spatial dynamics distribution along the entire rosette area, we defined five *circular rings* with equal width (i.e., width is rosette size-dependent) for each rosette, starting from rosette center and outwards, and quantified measures for each ring. We found that luminal and peripheral rings exhibited reduced radial organization, B/A ratio and speed, while peak levels were recorded within intermediate rings (S5 Fig). This observation was more prominently expressed for E-RG rosettes. The poor performance of cells in distal rings of M-RG rosettes support a model where distal cells in M-RG rosettes “suffer” from mixed populations comprised of more differentiated cells that have detached the lumen (apical sites) towards periphery (Fig 1A, bottom panel; Ref. [20]) and thus should reduce organized motion.

### Temporal analysis reveals that M-RG rosettes exhibit functional instability

**Fig 6:**
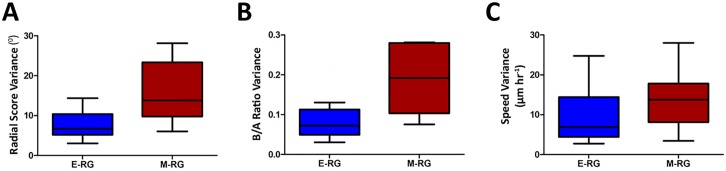
Functional instability of M-RG rosettes. Temporal variance of RS **(A)**, B/A **(B)** and Speed **(C)** was calculated for each rosette over time. **A.** E-RG rosettes are more stable in RS compared to M-RG rosettes (Wilcoxon rank sum test, p = 0.0009). **B.** E-RG rosettes are more stable in B/A compared to M-RG rosettes (Wilcoxon rank sum test, p = 0.0006). **C.** E-RG rosettes are more stable in Speed compared to M-RG rosettes (Wilcoxon rank sum test, p = 0.03). 25 E-RG rosettes and 14 M-RG rosettes were analyzed.

The reduced dynamics and the broader cellular heterogeneity of M-RG rosettes suggest that these rosettes are mechanically compromised, reflecting the progressive disassembly of rosettes, which culminates around day 55 [20]. We therefore tested whether rosette disassembly is reflected functionally in INM measures also at the shorter range of time, i.e. throughout the time-lapse experiment. This was quantified by calculating each measure for each rosette at every time point independently and then recording its temporal variance (Methods). Importantly, no temporal trend was observed during the 4-hour imaging course of an experiment (S2E Fig). This implies that the variance encodes the fluctuations in a certain rosette measure over the imaging time course, a measure we term *functional instability*. Indeed, temporal variance of RS, B/A and speed was significantly higher for M-RG rosettes (Fig 6), suggesting functional instability as an indicator for the stage of progression in rosette disassembly. Importantly, these high temporal variances observed for M-RG rosettes measure their reduced ability in consistently performing INM, arguably due to their compromised structure. They do not measure the actual rosette disassembly process, which occurs in a longer time scale (from day 14 to day 35, and culminating towards day 55). Taken together, our results validate functional instability as a reliable readout for rosette organized dynamics.

### Inhibition of ACTIN or NMII reduces INM

**Fig 7:**
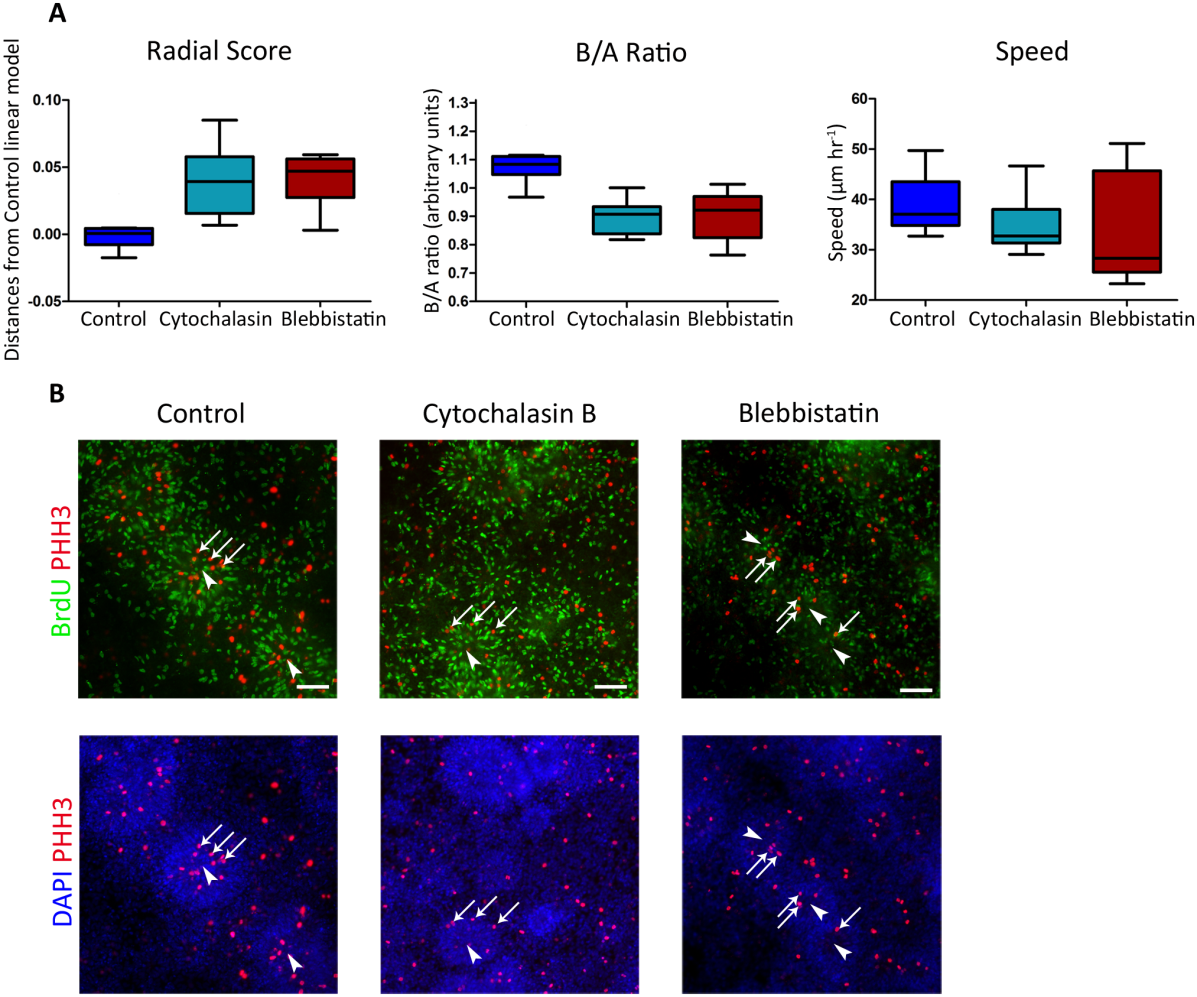
Inhibition of ACTIN or NMII reduces INM. Treatment with Blebbistatin or Cytochalasin-B reduces INM measures. E-RG rosettes were treated with or without indicated inhibitors immediately prior to live imaging and throughout the experiment. **A.** INM measures: Left: RS—signed distances of RS of treated rosettes from control E-G linear model (Blebbistatin: Wilcoxon rank sum test, p = 8.6841e-04; Cytochalasin-B: p = 1.5890e-04). Middle: B/A ratio (Blebbistatin: p = 3.1994e-04; Cytochalasin-B: p = 6.0475e-05). Right: Speed (Blebbistatin: p = 0.2370; Cytochalasin-B: p = 0.0434). Note that all measures were significantly reduced for drug-treated cells excluding speed for Blebbistatin-treatment. 10 control E-RG rosettes, 8 treated with Blebbistatin and 14 with Cytochalasin-B were analyzed in panels A-C. **B.** Treatment with Blebbistatin or Cytochalasin-B disrupts the ordered spatial distribution of cell cycle markers within rosettes. E-RG rosettes were labeled with BrdU immediately following the experiment and then fixed and immunostained for BrdU and PHH3 marking DNA replication (green) and mitosis (red) phases, respectively. DAPI marks nuclei. Sites of mitosis (PHH3+, arrows) are less confined to rosette centers (arrowheads) under inhibitor treatments.

Finally, to further strengthen the validity of our method and to shed some light on mechanisms of INM in vitro, we quantified the effects of pharmacological perturbation on INM. Different molecular motors are thought to mediate nuclei migration [12,34]. Such motors are believed to be a part of the cytoskeletal structural machinery such as actin, or motor proteins such as NMII, both shown to be involved in INM movements [35]. We treated rosettes with Blebbistatin or Cytochalasin-B, two agents known to alter INM by inhibiting NMII ATPase activity or depolymerizing actin, respectively. Quantifying INM dynamics in these rosettes following live imaging (S4–S6 Movies) showed decrease in INM measures (Fig 7A). This was further supported by demonstrating a loss of the ordered spatial composition of cell cycle components within rosettes. This was judged by immunostaining for mitosis (PHH3) and DNA synthesis (BrdU)–two distinct phases in the cell cycle that are spatially distributed to lumen and periphery, respectively (Fig 7B) (See also Ref. [20]). These findings further validate the ability of our quantitative approach to distinguish between rosettes under different molecular perturbations and further provide new evidence for possible roles of these molecular motors in driving INM in human radial glial cells.

## Discussion

Neural rosettes are highly organized multi-cellular structures that are formed by NSCs and are a cytoarchitectural hallmark during the transition of pluripotent stem cells into cortical fates in vitro. Here, we provided some initial insights and possible mechanisms for the complex dynamics of these structures, implicating intrinsic size-dependency of radial organization driven by mechanical constraints, inherently elevated radial organization for E-RG rosettes, and enrichment of basal motions as yet another potential contributor to enhanced and more stable INM dynamics of E-RG rosettes.

**Fig 8:**
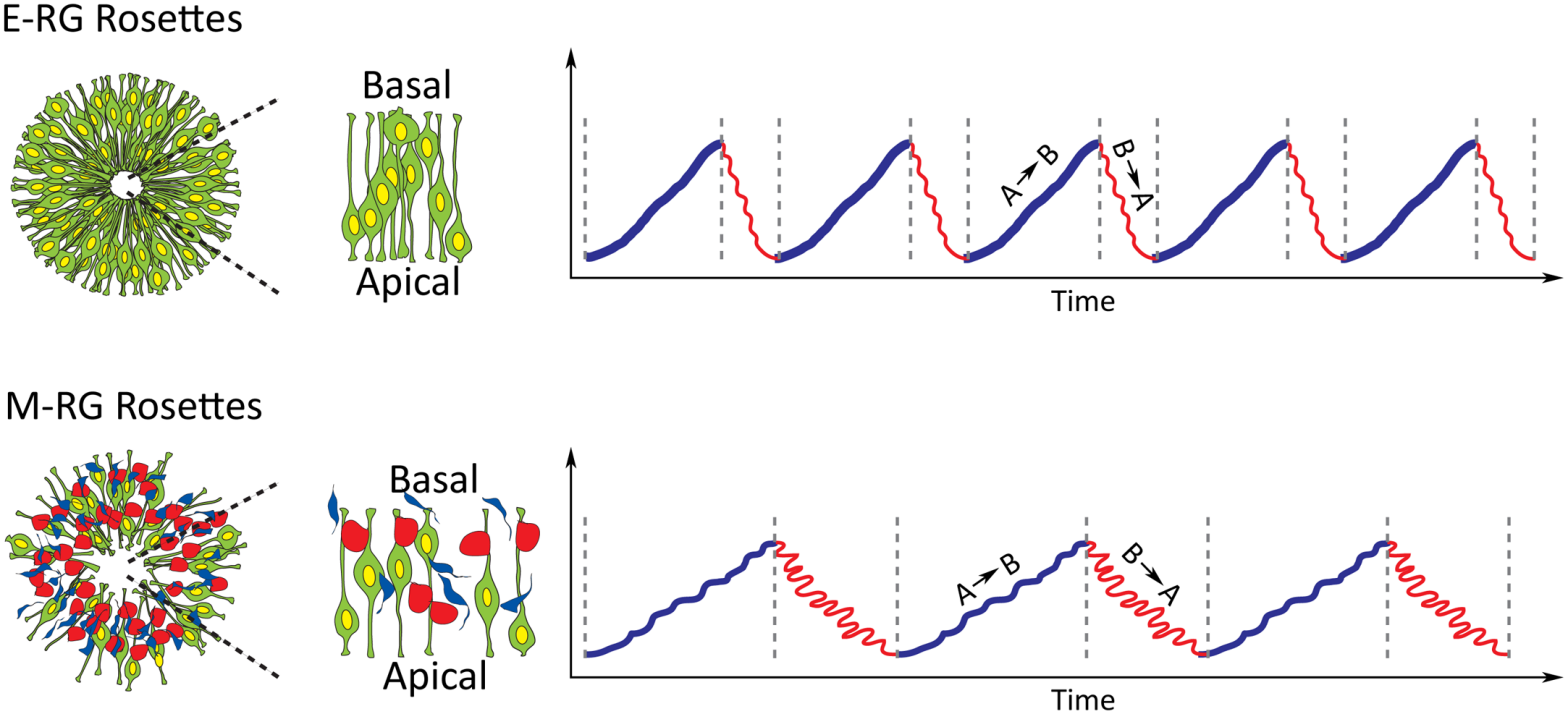
Cytoarchitectural dynamics of neural rosettes reflects changes in NSC capabilities during cortical development. E-RG rosettes (top panel) correspond to early cortical radial glial cells (NSCs; green colored) that hold strong epithelial characteristics and hence organize in a radial manner with apical sites adjoining at rosette lumens—similarly to the ventricular zone of the developing cortex. M-RG rosettes (bottom) are characterized by decreased numbers of epithelial radial glial cells and elevated number of neurons (blue colored) and intermediate progenitors (red colored), which are both non-epithelial—hence decreasing rosette epithelial integrity and eventually lead to rosette disassembly at later stages. Both E-RG and M-RG rosettes perform INM—the hallmark of cortical radial glial development. INM of E-RG rosettes is characterized by basal (blue phase, right) and apical (red phase, right) motions that are faster (higher frequency of blue and red phases) and more radially organized (less twisted pattern of blue and red phases), compared to M-RG rosettes. However, for all rosettes regardless of developmental stage (top or bottom panels), basal motions (blue) are always slower yet more organized than apical motions (red). The enhanced radial organization of E-RG rosettes can be explained by enhanced radial organization of basal motions as well as inherent mechanism that increases both basal and apical motions, possibly due to the strong confining structure and high NSC abundance within E-RG rosettes. B→A, basal to apical; A→B, apical to basal.

We propose three quantitative measures as means to quantify rosette dynamics: RS, B/A ratio and speed. These measures were used to assess differences in dynamics between early (E-RG) and late (M-RG) rosettes, revealing that INM of early rosettes is more efficient. This may well reflect the situation in the developing cortex: E-RG rosettes correspond to symmetrically dividing NSCs during early cortical development [20], which exhibit high self-replication rates and low levels of differentiation, resulting in increased number of cells undergoing INM within the ventricular zone. This implies that radial glial cells during early cortical development hold inherently elevated radial organization that may be required for accommodating the high traffic and orchestrating cell motion and cell division. In this regard, the radial expansion of the ventricular zone, which can be mirrored in vitro by the emergence of larger rosettes, may add greater mechanical constrains that ultimately contribute as well to enhance radial organization. At more advanced stages of cortical development, which are reflected by the M-RG stage in vitro [20], the production of neurons and intermediate progenitors—both non-polarized cells—is prevalent due to increase in asymmetric cell division of the corresponding radial glial cells. This occurs on the expanse of polarized NSCs adjacent to apical sites, which still perform INM. Thus, the accumulation of differentiated progeny increases non-NSC ratios, which in turn disrupt radial organization performance (Fig 8). To conclude, our analyses provide a first link between function and dynamics.

Literature survey demonstrated notable similarity between speeds measured across different labeling methods, in different species, in vivo and in vitro. Tsai et al (Ref. [16]) used cytoplasmic GFP for INM quantification of radial glial cells in mouse cortical slices ex vivo and shows higher speeds for apically directed motions (up to a 60μm/hour) compared to basally directed motions (up to 30 μm/hour). An in vivo study in zebra fish (Ref. [33]) shows comparable results using the more classic nuclei labeling, with speed of up to 20μm/hour and 3.4μm/hour for apical and basal motions, respectively. Similarly, although to a different extent, our in vitro findings show that human E-RG rosettes corresponding to early developing cortical radial glial cells exhibit faster speed for apical motions (38.8μm/hour) compared to speed of basal motions (35.3μm/hour), while the more advanced M-RG rosettes corresponding to mid neurogenesis moved apically at 30.3μm/hour and basally at 27.1μm/hour. This agreement is served as additional means to increase our confidence of using neural rosettes as physiologically relevant in vitro model system for cortical expansion as well as validating the capabilities of our analysis to quantify this process.

Our encouraging results suggest that this analytical framework may enable high-content quantification for diagnosis, molecular investigation and drug screening. Recent advancements in the stem cell field allow obtaining skin biopsies from patients and convert them into pluripotent stem cells (induced pluripotent stem cells (iPS cells)) [36]. Such iPS cells can be derived from patients with cortical diseases and then re-directed from the pluripotent stage into neural rosettes. We envisage that applying these quantitative measures on rosettes derived from patient iPS cells will have the potential to reflect damaged or genetic mutation-affected properties of NSCs. In addition, the effects of a specific drug / molecular perturbation could be predicted based on its alternation on these measures. Thus, this could be the first step toward developing platforms for understanding rosette dynamics in health and disease.

## Methods

### Ethics statement

We used the human embryonic stem cell line WA-09 (H9) purchased from WiCell. Tel Aviv University Ethics Committee (IRB) approved the usage of existing human embryonic stem cell lines including H9. Material transfer agreement (MTS) was signed between Tel Aviv University (Vice President for R&D) and WiCell with regard to the transfer and usage of the human ES cell line WA09 in the Elkabetz lab, and following Agreement Letter between the Principal Investigator and WiCell. The use of human embryonic stem cells for therapeutic research is allowed in Israel. For more details see text on "The Use of Embryonic Stem Cells for Therapeutic Research", available at http://bioethics.academy.ac.il/english/report1/report1-e.html.

### Culturing undifferentiated hESCs

The human ES cell (hESC) line H9 (WA-09; Wicell—Wisconsin) and the H9-derived BAC transgenic *HES5*::*eGFP* line [22] were cultured on mitotically inactivated mouse embryonic fibroblasts (MEFs) (Globalstem). Undifferentiated hESCs were maintained as described previously [17] in medium containing DMEM/F12, 20% KSR, 1mM Glutamine, 1% Penicillin/Streptomycin, non-essential amino acids and beta-mercaptoethanol.

### Neural induction, and rosette formation and long-term culture

Experiments were performed as described in Refs. [20,21]. For neural induction and generation of neuroepithelial and radial glial cells, hESC colonies were removed from MEFs by Dispase (6U/ml, Worthington), dissociated with Accutase (Innovative Cell Technologies, Inc.), plated at sub confluent cell density (40-50K cells/cm^2^, although twice higher density or alternatively small hESC clusters work well and accelerate confluence) on Matrigel (1:20, BD) coated dishes, and supplemented with MEF-conditioned media and 10μM ROCK inhibitor (Y-27632, Tocris) with daily fresh FGF2 (10 ng/ml, R&D). Confluent cultures were subjected to dual SMAD inhibition neural differentiation using Noggin (R&D, 250 ng/ml) and SB-431542 (10 μM, Tocris), and further supplemented with LDN-193189 (100 nM, Stemgent) (denoted LNSB protocol). HES5::eGFP usually appears on day 8 or 9 of neural differentiation. To generate E-RG rosettes and subsequent progenitors, NE cells were scrapped from plates on day 10–12, pre-incubated with Ca^+2^/Mg^+2^ free HBSS followed by collagenase II (2.5 mg/ml), Collagenase IV (2.5 mg/ml) and DNAse (0.5 mg/ml) solution (all from Worthington) (37 degrees, 20 minutes). Cells were then dissociated and replated at high density (500,000 cells/cm^2^) on moist matrigel drops, and grown for additional days till rosettes appeared (E-RG stage). Neural induction and direct formation of E-RG stage rosettes could be also formed by co-culture of hESC clusters with MS5 stromal cells as previously described [17]. Briefly, early appearing rosettes on MS5 were harvested mechanically beginning on day 8–10 of differentiation, replated on culture dishes pre-coated with 15 μg/mL polyornithine (Sigma), 1 μg/mL Laminin (BD Biosciences) and 1 ug/ml Fibronectin (BD Biosciences) (Po/Lam/FN) till Day 14, to obtain E-RG rosettes. Under both protocols, early appearing NE cells were cultured from Day 9 with N2 medium (composed of DMEM/F12 and N2 supplement containing Insulin, Apo-transferin, Sodium Selenite, Putrecine and Progesterone), and further supplemented with low SHH (30ng/mL), FGF8 (100ng/mL) and BDNF (5ng/mL). Long-term culture of E-RG rosettes was performed by a weekly mechanical harvesting of rosettes and re-plating on Po/Lam/FN coated dishes with N2 medium, SHH and FGF8, till Day 28. These were replaced by FGF2 (20ng/mL) and EGF (20ng/mL) on Day 28 (all cytokines from R&D Systems). At day 35, E-RG rosettes reached the M-RG rosette stage. Cells were replated as clusters from one passage to another to reach the M-RG stage.

### Immunostaining, confocal LSM and live imaging

Cells were fixed in 4% paraformaldehyde, 0.15% picric acid, permeabilized and blocked with PBS, 1% FBS and 0.3% Triton solution, and stained with indicated primary antibodies followed by AlexaFluor secondary antibodies (Invitrogen). Cells were imaged in PBSx1 after staining. All cell imaging was carried out in 24 well glass bottom plates (In Vitro scientific). Fluorescence images were obtained using a Nikon Eclipse Ti-E microscope or a confocal LSM710 microscope (Carl Zeiss MicroImaging, Germany). The still or time-lapse images were captured using a 10x and a 20× objectives (NA = 0.3, 0.8 respectively, Plan-Apochromat).

Fluorescence emissions for eGFP, CY3, CY5 and DAPI channels were detected using filter sets supplied by the manufacturer.

For live imaging, cultured cells were maintained on the microscope stage in a temperature, CO2, and humidity-controlled environmental chamber. Time-lapse eGFP and phase matched contrast images were acquired using Nikon Eclipse Ti-E microscope every 5 minutes for over 4 hours (250 minutes). Physical pixel size was 0.64 x 0.64 μm. Images and movies were generated and analyzed using the NIS elements software (Nikon). All images were exported in TIF and then processed by our quantitative tools. 25 E-RG rosettes and 14 M-RG rosettes were live imaged in N = 2 independent experiments and quantified as detailed below.

### Molecular perturbations

E-RG rosette cells were treated with either Blebbistatin (5μM, Sigma) or Cytochalasin-B (0.5μg/ml, Sigma) and concomitantly recorded for 250 minutes as described above. 10 control E-RG rosettes, 8 E-RG rosettes treated with Blebbistatin and 14 E-RG rosettes treated with Cytochalasin-B were live imaged and analyzed.

### Quantitative analysis of live imaging of neural rosettes

Rosette centers and contours were manually annotated from the phase contrast channel, where the cytoarchitecture outlines of the region performing INM become obvious to a human eye based on different texture-patterns in the image. Rosette centers and peripheries were marked independently based on subjective identification of regions performing INM as reflected in the image-texture of the phase-contrast channel. Rosette contours were manually validated to remain stable throughout the time-lapse images (S1B Fig), in accordance with the different time scales of rosette imaging and rosette disassembly (S2E Fig). Independent annotations showed highly similar kinetic measures (S6 Fig). The expected radial angle was calculated in relation to the marked center for every location within the region-of-interest defined by the rosette contours. *Rosette size* was defined as the diameter of the circle that best fits the rosette-annotated contour. Local motion estimation was extracted by maximizing local cross correlation as described in Ref. [24]. Briefly, given two consecutive *HES5*::*eGFP* fluorescence images *t*, *t*+1 from the time-lapse sequence *(i)* Partition the current image (at time *t*) to a grid of sub-cellular sized local patches, of size 8.3μm x 8.3μm each (13 x 13 pixels); *(ii)* Find maximal cross-correlation to the next frame (*t*+1) to retrieve the local motion estimations for each patch. The search radius was defined based on maximal speed of 120μm / hour; *(iii)* Extract velocity angles and magnitude (speed) from the local motion estimation for each patch; *(iv)* Exclude motions below 15 μm hr^-1^ from all measures calculations. The quantized motion angles for each patch in the rosette were recorded for 250 minutes (50 frames). For each patch at every time point the following two angles were defined: *Expected radial angle* is the orientation of the vector between the rosette center and the patch at hand. The *angular alignment* γ of a given patch’s motion at a given time is the angle between the local velocity angle and its corresponding expected radial direction (Fig 2). A measure for *radial organization* was defined as the average angular alignment across all patches over time. This measure was termed *Radial Score* (RS), were high values reflected poor radial organization throughout a time-lapse experiment. Only patches that move at speed above 15 μm hour^-1^ (≥ 2 pixel per frame) were considered for calculating rosette radial organization, because small motions limit the discretization of the velocity angles which cause unreliable high angular deviations. The same minimal motion was considered for the rest of the analysis.

The velocity of each patch at every time was classified as apical or basal based on its direction in relation to the rosette center (Fig 2). Velocity angles pointing toward the rosettes periphery (+/- 90 degrees) were classified as basal, while toward the rosette center (+/- 90 degrees) as apical. The ratio of all basal to apical motions was termed *basal-to-apical ratio (B/A ratio)*, where value of 1 reflects equal numbers of patches’ motions moving apically and basally, values > 1 corresponds to more motion toward the rosette periphery. RS for basal (correspondingly, apical) motions were calculated exactly as described above, only considering basal (apical) motions.

The measure *Speed* was defined as the average magnitude of velocity across all patches over time (Fig 2). Higher values reflect faster average motion throughout a time-lapse experiment. Basal or apical speed was calculated by considering the average of solely the basal or apical motions, respectively.

The variance for RS, B/A ratio and speed was calculated over time. Each of the measures was calculated independently for each frame in the time-lapse experiment (one scalar readout per frame), and the variance was recorded.

For spatial analysis, 5 different regions, at growing distances-intervals from the rosette center were defined for each rosette. These regions, termed *circular rings*, were each analyzed independently throughout the time-lapse experiment: all patches in each ring over time were used to calculate RS, B/A ratio and speed. Since rosette geometry was not a perfect circle, the last ring was not always complete, but confined by the rosette contour. Note that the width of a circular ring was rosette specific and changed as function of rosette size.

### Statistics

The nonparametric Wilcoxon rank sum test was used to assess statistical significance between E-RG and M-RG rosettes and for the perturbation experiments (Matlab function ranksum). Nonparametric Wilcoxon sign rank test was used to assess statistical significance between two measures calculated independently on the same rosette (e.g., apical vs. basal measures, Matlab function signrank). Pearson’s linear correlation was used to calculate associations and their corresponding p-values (Matlab function corr). Least square fit was applied to calculate the linear models that best fits the data (Matlab function polyfit). Box plots: solid black line inside the box is the median, bottom and top of the box are the 25% and 75% percentile, respectively.

## Supporting Information

S1 Note Enrichment of basal motions is suggested as a secondary mechanism for elevated organization of E-RG rosettes. (DOCX)

S1 Movie Radial patterns of cell dynamics in E-RG rosettes. Time-lapse images of *HES5*::*eGFP* **(first time-lapse)** and their phase construct-matched images **(consecutive time-lapse)** for E-RG rosettes. Images were captured for over 4 hours (250 min) at 5-min intervals as described in Methods. Annotation for E-RG rosettes is shown in Fig 1B. For analysis purposes, raw data series were exported in TIF format, as GFP and Phase separately. Scale bar: 50 μm.
(MOV)

S2 Movie Radial patterns of cell dynamics in non-Rosettes. Time-lapse images of *HES5*::*eGFP* **(first time-lapse)** and their phase construct-matched images **(consecutive time-lapse)** for Non-rosettes. Images were captured for over 4 hours (250 min) at 5-min intervals as described in Methods. Annotation for Non-Rosettes is shown in Fig 1B. For analysis purposes, raw data series were exported in TIF format, as GFP and Phase separately. Scale bar: 50 μm.
(MOV)

S3 Movie Radial patterns of cell dynamics in M-RG Rosettes. Time-lapse images of *HES5*::*eGFP* **(first time-lapse)** and their phase construct-matched images **(consecutive time-lapse)** for M-RG rosettes. Images were captured for over 4 hours (250 min) at 5-min intervals as described in Methods. Annotation for M-RG rosettes is shown in S1A Fig. For analysis purposes, raw data series were exported in TIF format, as GFP and Phase separately. Scale bar: 50 μm.
(MOV)

S4 Movie Radial patterns of cell dynamics in Cytochalasin-B treated E-RG Rosettes. Time-lapse images of *HES5*::*eGFP* **(first time-lapse)** and their phase construct-matched images **(consecutive time-lapse)** for E-RG rosettes treated with Cytochalasin-B. Images were captured immediately following inhibitor administration for over 4 hours (250 min) at 5-min intervals as described in Methods. For analysis purposes, raw data series were exported in TIF format, as GFP and Phase separately. Scale bar: 50 μm.
(MOV)

S5 Movie Radial patterns of cell dynamics in Blebbistatin treated E-RG Rosettes. Time-lapse images of *HES5*::*eGFP* **(first time-lapse)** and their phase construct-matched images **(consecutive time-lapse)** for E-RG rosettes treated with Blebbistatin. Images were captured immediately following inhibitor administration for over 4 hours (250 min) at 5-min intervals as described in Methods. For analysis purposes, raw data series were exported in TIF format, as GFP and Phase separately. Scale bar: 50 μm.
(MOV)

S6 Movie Radial patterns of cell dynamics in Control E-RG Rosettes. Time-lapse images of *HES5*::*eGFP* **(first time-lapse)** and their phase construct-matched images **(consecutive time-lapse)** for E-RG rosettes without any inhibitors used as control for the perturbation experiments. Images were captured for over 4 hours (250 min) at 5-min intervals as described in Methods. For analysis purposes, raw data series were exported in TIF format, as GFP and Phase separately. Scale bar: 50 μm.
(MOV)

S1 Fig Rosette manual annotation. **A.** Annotation of M-RG rosettes. Top: Representative *HES5*::*eGFP* (left) and its matched phase contrast image (right) from time-lapse imaging of an M-RG stage neural rosette. Rosette contours and center were manually annotated (white dashed marking) based on the phase contrast channel. Many radial glial cells at the M-RG stage delaminate the lumen toward rosette periphery but still retain GFP for some time. These correspond to non-epithelial progenitors and hence should be excluded as achieved by annotation based on the phase contrast channel. Bottom left: Motion patterns follow the expected radial angle. Average patch velocity orientation over time for M-RG rosette. Bottom right: Distributions of angular alignment of all patches over the entire time course. INM patterns are observed for M-RG rosettes (mean angle of 30°). Scale bars: 25 μm. **B.** Rosette contours remain stable over time. Phase contrast images of E-RG (top) and M-RG (bottom) rosettes and their corresponding manual annotations (white dashed marking). Rosette structure remains stable (same center location and contours, X and Y; dashed red lines) during the imaging time-course (shown t = 0, 250 minutes). Scale bars: 25 μm.
(TIF)

S2 Fig Technical aspects in rosettes quantification. **A.** Rosette discretization to sub-cellular patches. Rosette contours were manually annotated (white dashed contour), and discretized to a grid of subcellular patches (upper-left). Zoom into a region shows GFP data of 6x6 patches region (upper-right). Zoom into a single patch containing 13x13 pixels shows the resolution used to estimate the motion vectors by matching texture patterns over time (bottom-right). **B.** Patch-based motion estimation is correlative to manual single-cell tracking. High correlation is shown between patch-based motion estimation (used in our framework, here denoted as PIV) and single cell manual tracking as ground truth. The former approach was selected to allow robustness and high-throughput quantification that do not depend on accurate single cell segmentation. RS (left, Pearson rho = 0.7528, pval = 1.5487e-23) and speed (right, Pearson rho = 0.7041, pval = 1.4669e-19). **C.** Distribution of patches speed for a representative E-RG (left) and M-RG (right) rosette. Speeds below 15μm hour^-1^ were excluded from measures calculations and further analysis. Note that including speeds below the threshold would make the difference in speed between E-RG and M-RG rosettes even more extreme than presented in Fig 5, because there are more slow motions in M-RG rosettes. **D.** Normal distribution of velocity orientations with mean at the radial expected angle. Image patches were partitioned to 8 radial groups based on their expected radial angle (intervals of 22.5 degrees, numbered as 1 to 8 as indicated in the schematic sketch of angular alignment at the bottom panel). Each displayed distribution was calculated for all observed velocity angles over time for each of these 8 radial groups separately. Top, left-to-right: radial groups 1–4 (representing 0–22.5, 22.5–45, 45–67.5 and 67.5–90 degrees) are on, bottom, left-to-right: radial groups 5–8 (representing 90–112.5, 112.5–135, 135–157.5 and 157.5–180 degrees). For each distribution (y-axis), x-axis represents motion within each of these radial groups. Note that (1) these distributions are circular, e.g., group 1 is most similar to group 2 and 8, and (2) motions within 0–180 and within 180–360 degrees are collapsed (e.g., motions within 0–22.5 degrees range include also motions within 180–202.5 degrees range (and both are in radial group 1). Normal distributions were observed for all radial groups with mean at the expected radial angle. The Analysis was performed on a representative E-RG rosette (top) and similar distributions were replicated for the phase-contrast channel (middle). **E.** Rosette measure fluctuates over imaging time-course. Distributions of the slope of RS (left), B/A ratio (middle) and speed (right) over time for E-RG and M-RG rosettes. Each measure was calculated for each rosette over time, the slope of its linear fit was recorded and the distribution of all rosettes’ slopes was presented. The rationale was that a trend in the data (e.g., rosette RS increases during the imaging course) would be reflected in a corresponding slope different than zero. The slope distributions seem to be around values of zero suggesting that no temporal trend is present within the imaging course. This data validates that the four-hour imaging course is not reflecting the progressive rosette-disassembly in culture from E-RG rosette formation on day 14 to partial disassembly of M-RG rosettes on day 35 to complete rosette disassembly around day 55. Thus, this observation allows us to consider mean measures of accumulation over time as our readouts.
(TIF)

S3 Fig Enhanced B/A ratio contributes elevated radial organization of E-RG rosettes. **A.** B/A ratio. The number of basal motions mostly outnumbered the number of apical motions within rosettes (left, above the black y = x line, Wilcoxon sign rank test: E-RG, p = 1.229E-05; M-RG, p = 0.0353). Bottom panel: enlarged version of the marked box shown on the top panel to make partially-overlapping data points visible. Right, B/A ratios were higher for E-RG rosettes compared to M-RG rosettes, as calculated by t-test (p = 0.016, assume normality), but not as significantly (right, Wilcoxon rank sum test, p = 0.1106). **B.** E-RG rosettes exhibit higher correlation between RS and B/A ratios compared to M-RG rosettes (Pearson, Rho = -0.5958, p = 0.0017; MRG Pearson, Rho = -0.3653, p = 0.199). **C.** B/A ratios were not found to be associated with rosette size (Pearson, E-RG Rho = 0.2957, p = 0.1512; M-RG Rho = 0.1081, p = 0.7130). **D.** Differences between RS of apical and basal motion are associated to B/A ratios (Pearson, E-RG Rho = 0.6396, p = 5.762E-04; M-RG Rho = 0.7767, p = 0.0011). Solid lines are the linear fit for E-RG and M-RG data. **E.** Differences between RS of apical and basal motion are associated to RS of basal motion (left, Pearson, E-RG Rho = -0.4975, p = 0.0114; M-RG Rho = -0.5536, p = 0.04), but are not associated with RS of apical motion (right, Pearson, E-RG Rho = -0.1924, p = 0.3569; M-RG Rho = -0.1326, p = 0.6512). Solid lines are the linear fit for E-RG and M-RG data. 25 E-RG rosettes and 14 M-RG rosettes were analyzed.
(TIF)

S4 Fig Rosette speed was not found to be associated with rosette size. Pearson: E-RG: overall speed: Rho = 0.3728, p = 0.0664, E-RG basal speed: Rho = 0.3559, p = 0.0808, E-RG apical speed: Rho = 0.3638, p = 0.0738, M-RG: overall speed: Rho = 0.4484, p = 0.1078, M-RG basal speed: Rho = 0.2774, p = 0.3370, M-RG apical speed: Rho = 0.5438, p = 0.0444. Linear fit: dashed line for apical, solid for basal motion. 25 E-RG rosettes and 14 M-RG rosettes were analyzed.
(TIF)

S5 Fig Spatial dynamics of neural rosettes. 5 circular rings with equal widths are defined from each rosette center. Ring #1 is closest to the rosette center while #5 to the periphery. E-RG (left) versus (M-RG) for RS **(A)**, Speed **(B)** and B/A **(C)**. 25 E-RG rosettes and 14 M-RG rosettes were analyzed.
(TIF)

S6 Fig Manual annotation of rosettes is consistent and shows highly similar kinetic measures. Assessment of the variability in kinetic measures caused by different manual rosette annotations. Three independent manual annotations of rosette centers and contours were used to calculate RS **(A)**, B/A ratio **(B)** and speed **(C)**. In each panel, x-axis represents the measure of the first annotation that was used to calculate the measures reported in Figs 3–7, y-axis displays the measures of the two additional annotations. The black line Y = X is used as guideline. Pearson correlation was used for statistics comparing 3 pairs of annotations for each measure. **A.** RS: Rho = 0.8773, p = 8.6006e-09; Rho = 0.8605, p = 3.4538e-08; Rho = 0.9610, p = 2.4466e-14. **B.** B/A ratio: Rho = 0.9151, p = 1.5085e-10; Rho = 0.9147, p = 1.5845e-10; Rho = 0.9531, p = 1.9627e-13. **C.** Speed: Rho = 0.9812, p = 6.0661e-18; Rho = 0.9844, p = 7.4069e-19; Rho = 0.9807, p = 8.4915e-18.
(TIF)
