# Supplementary material for: Quantitative Live Imaging of Human Embryonic Stem Cell Derived Neural Rosettes Reveals Structure-Function Dynamics Coupled to Cortical Development
Source: PLoS Comput Biol. 2015 Oct 16;11(10):e1004453. doi: 10.1371/journal.pcbi.1004453 (PMC4608579; doi:10.1371/journal.pcbi.1004453)
Supplement: S1 Note — (DOCX) [file pcbi.1004453.s001.docx]

**S1 Note**

**Enrichment of basal motions is suggested as a secondary mechanism for elevated organization of E-RG rosettes**

The results presented in **Fig 4** led to the hypothesis that enhanced radial organization of basal motions contributes to the overall elevated radial organization observed for E-RG rosettes. We next found that basal motions (that tend to be more organized) outnumbered apical motions (that tend to be less organized) **(S3A Fig, left, B/A ratios > 1 for most rosettes).** These B/A ratios were higher for E-RG rosettes compared to M-RG rosettes, although not significantly **(S3A Fig, right)**. However, the distribution of B/A ratios was clearly extended compared to those of E-RG rosettes, speculatively projecting the higher variability in stage of rosette disassembly, as expected for longer time in culture. In accordance with these observations, E-RG rosettes displayed better correlation between B/A ratios and RS values **(S3B Fig)**. Importantly, B/A ratios were not associated with rosette size **(S3C Fig)**. This suggests that elevated B/A ratios in E-RG rosettes may contribute to increased radial organization in a size-independent manner, either by inherently affecting basal and apical RS, and/or by enhancing the contribution of basal motions, which are more radially organized than apical motions.

Additional evidence for a role for basal motion in mediating radial organization was found when explicitly quantifying the subtraction of basal from apical RS values. High levels of this measure reflect better organization of basal motions compared to that of apical motions. We found that this measure is linearly associated with B/A ratios **(S3D Fig)**, indicating that enhanced organization of basal motions coincide with increased number of basal over apical (B/A) motions. Interestingly, this plot further shows that when apical and basal RS become equal, the number of basal and apical motions equalizes (B/A ≈ 1) **(S3D Fig, intersection with x-axis)**. This observation suggests that radial organization is directly linked to the ratio of basal to apical motion. Finally, this difference in apical over basal RS was found to be associated with RS of basal motions, but not with RS of apical motions **(S3E Fig, compare left to right)**. This further suggests that the association between radial organization and the ratio of basal to apical motion can be explained by enhanced organization of basal motions rather than reduced organization of apical motions. Together, these results suggest that larger B/A ratios serve as a mechanism to enhance radial organization in E-RG rosettes by increasing contribution of basal motions. Yet, a more dominant, inherent mechanism likely exists for E-RG rosettes, which contributes to increase radial organization of both basal and apical motions.
