# Supplementary figures and images for: Quantitative Live Imaging of Human Embryonic Stem Cell Derived Neural Rosettes Reveals Structure-Function Dynamics Coupled to Cortical Development
Source: PLoS Comput Biol. 2015 Oct 16;11(10):e1004453. doi: 10.1371/journal.pcbi.1004453 (PMC4608579; doi:10.1371/journal.pcbi.1004453)

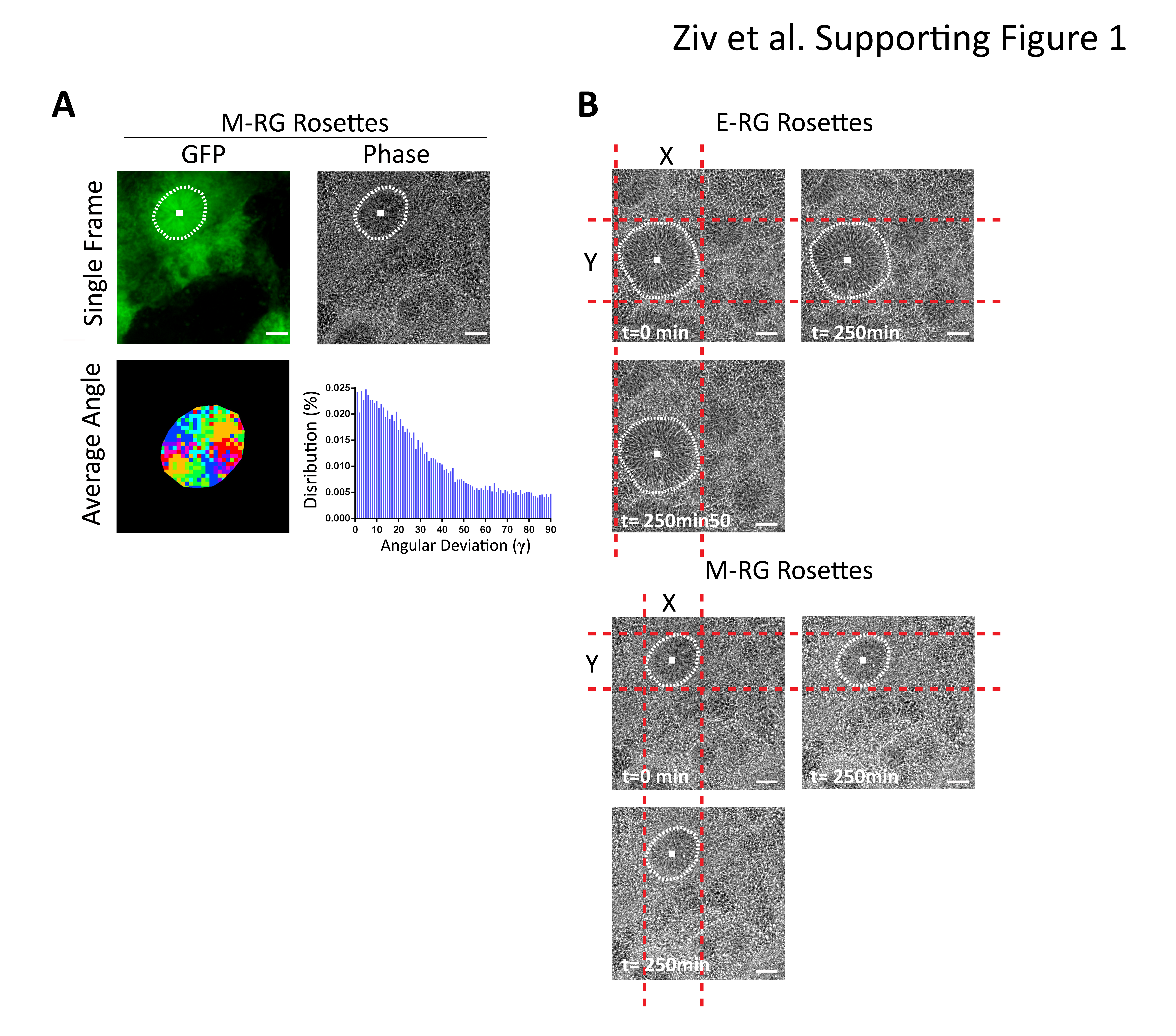

Supplement: S1 Fig — A. Annotation of M-RG rosettes. Top: Representative HES5::eGFP (left) and its matched phase contrast image (right) from time-lapse imaging of an M-RG stage neural rosette. Rosette contours and center were manually annotated (white dashed marking) based on the phase contrast channel. Many radial glial cells at the M-RG stage delaminate the lumen toward rosette periphery but still retain GFP for some time. These correspond to non-epithelial progenitors and hence should be excluded as achieved by annotation based on the phase contrast channel. Bottom left: Motion patterns follow the expected radial angle. Average patch velocity orientation over time for M-RG rosette. Bottom right: Distributions of angular alignment of all patches over the entire time course. INM patterns are observed for M-RG rosettes (mean angle of 30°). Scale bars: 25 μm. B. Rosette contours remain stable over time. Phase contrast images of E-RG (top) and M-RG (bottom) rosettes and their corresponding manual annotations (white dashed marking). Rosette structure remains stable (same center location and contours, X and Y; dashed red lines) during the imaging time-course (shown t = 0, 250 minutes). Scale bars: 25 μm. (TIF) [file pcbi.1004453.s008.tif]

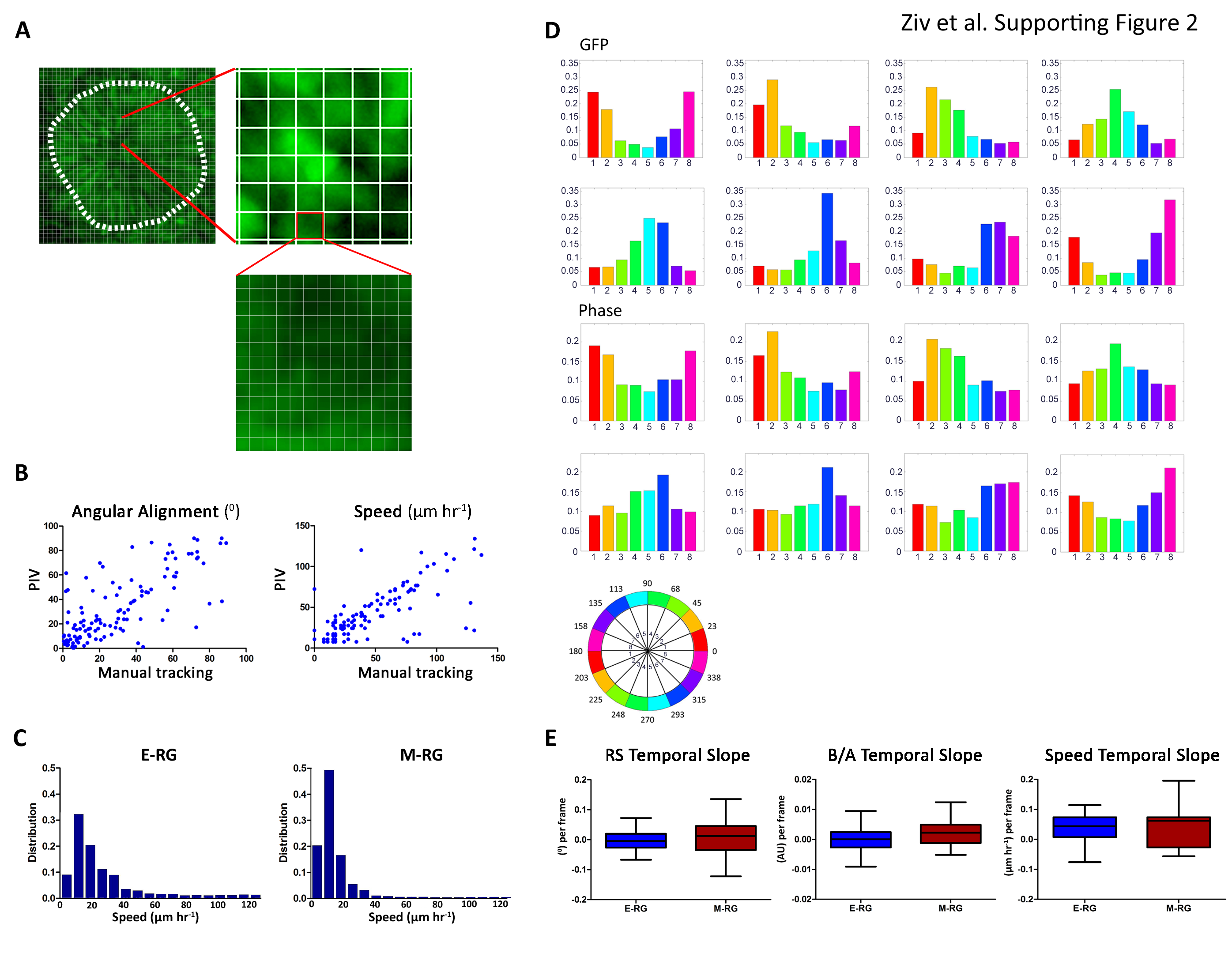

Supplement: S2 Fig — A. Rosette discretization to sub-cellular patches. Rosette contours were manually annotated (white dashed contour), and discretized to a grid of subcellular patches (upper-left). Zoom into a region shows GFP data of 6x6 patches region (upper-right). Zoom into a single patch containing 13x13 pixels shows the resolution used to estimate the motion vectors by matching texture patterns over time (bottom-right). B. Patch-based motion estimation is correlative to manual single-cell tracking. High correlation is shown between patch-based motion estimation (used in our framework, here denoted as PIV) and single cell manual tracking as ground truth. The former approach was selected to allow robustness and high-throughput quantification that do not depend on accurate single cell segmentation. RS (left, Pearson rho = 0.7528, pval = 1.5487e-23) and speed (right, Pearson rho = 0.7041, pval = 1.4669e-19). C. Distribution of patches speed for a representative E-RG (left) and M-RG (right) rosette. Speeds below 15μm hour-1 were excluded from measures calculations and further analysis. Note that including speeds below the threshold would make the difference in speed between E-RG and M-RG rosettes even more extreme than presented in Fig 5, because there are more slow motions in M-RG rosettes. D. Normal distribution of velocity orientations with mean at the radial expected angle. Image patches were partitioned to 8 radial groups based on their expected radial angle (intervals of 22.5 degrees, numbered as 1 to 8 as indicated in the schematic sketch of angular alignment at the bottom panel). Each displayed distribution was calculated for all observed velocity angles over time for each of these 8 radial groups separately. Top, left-to-right: radial groups 1–4 (representing 0–22.5, 22.5–45, 45–67.5 and 67.5–90 degrees) are on, bottom, left-to-right: radial groups 5–8 (representing 90–112.5, 112.5–135, 135–157.5 and 157.5–180 degrees). For each distribution (y-axis), x-axis represents motio [file pcbi.1004453.s009.tif]

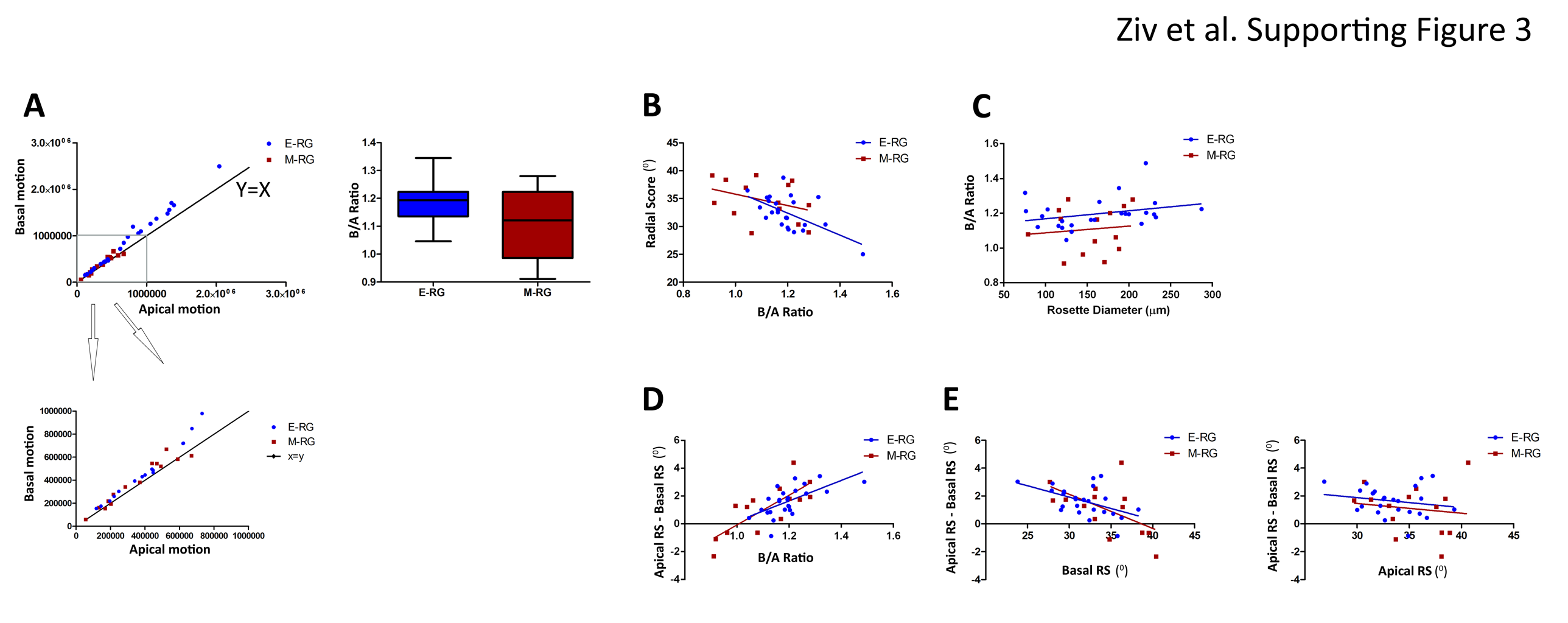

Supplement: S3 Fig — A. B/A ratio. The number of basal motions mostly outnumbered the number of apical motions within rosettes (left, above the black y = x line, Wilcoxon sign rank test: E-RG, p = 1.229E-05; M-RG, p = 0.0353). Bottom panel: enlarged version of the marked box shown on the top panel to make partially-overlapping data points visible. Right, B/A ratios were higher for E-RG rosettes compared to M-RG rosettes, as calculated by t-test (p = 0.016, assume normality), but not as significantly (right, Wilcoxon rank sum test, p = 0.1106). B. E-RG rosettes exhibit higher correlation between RS and B/A ratios compared to M-RG rosettes (Pearson, Rho = -0.5958, p = 0.0017; MRG Pearson, Rho = -0.3653, p = 0.199). C. B/A ratios were not found to be associated with rosette size (Pearson, E-RG Rho = 0.2957, p = 0.1512; M-RG Rho = 0.1081, p = 0.7130). D. Differences between RS of apical and basal motion are associated to B/A ratios (Pearson, E-RG Rho = 0.6396, p = 5.762E-04; M-RG Rho = 0.7767, p = 0.0011). Solid lines are the linear fit for E-RG and M-RG data. E. Differences between RS of apical and basal motion are associated to RS of basal motion (left, Pearson, E-RG Rho = -0.4975, p = 0.0114; M-RG Rho = -0.5536, p = 0.04), but are not associated with RS of apical motion (right, Pearson, E-RG Rho = -0.1924, p = 0.3569; M-RG Rho = -0.1326, p = 0.6512). Solid lines are the linear fit for E-RG and M-RG data. 25 E-RG rosettes and 14 M-RG rosettes were analyzed. (TIF) [file pcbi.1004453.s010.tif]

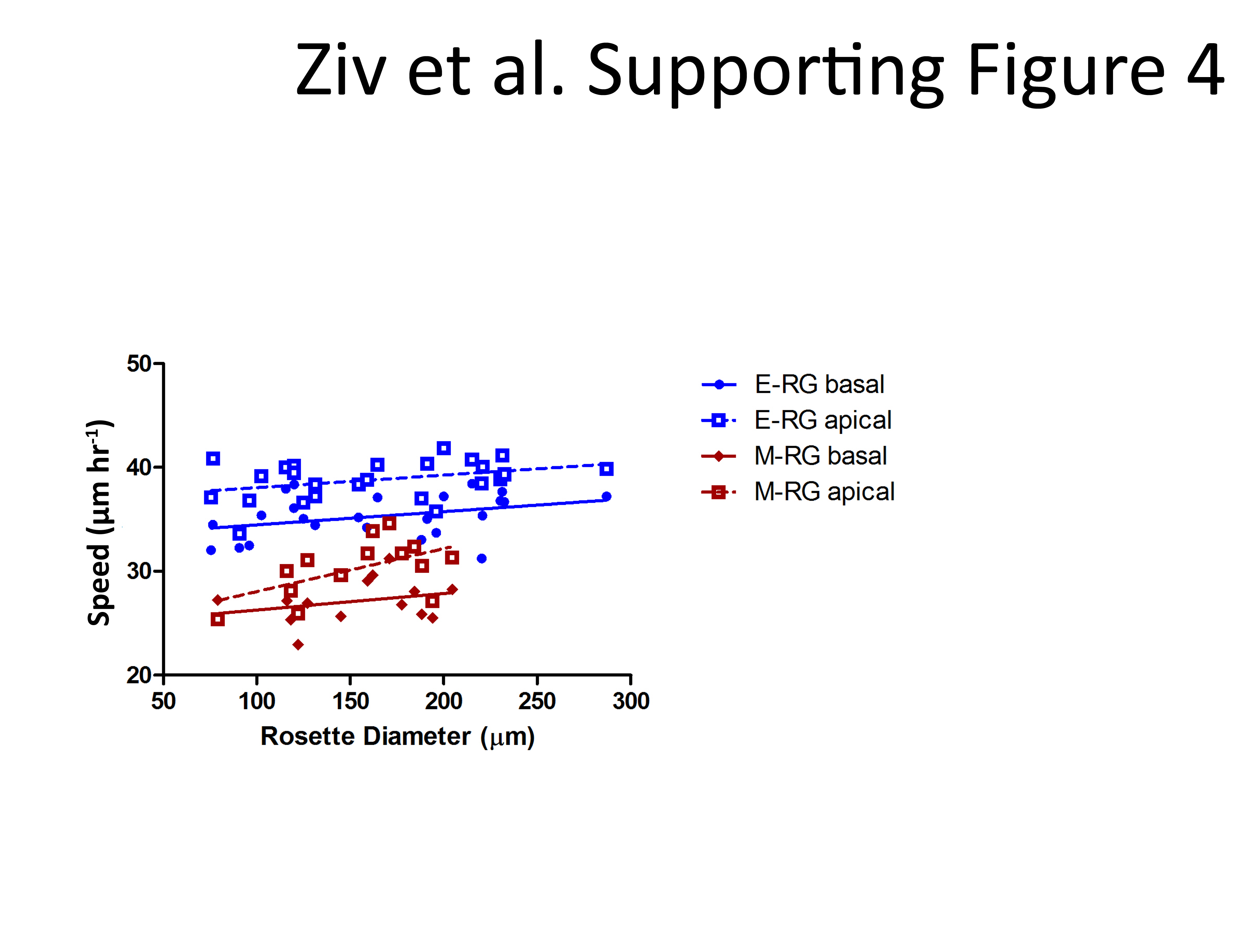

Supplement: S4 Fig — Pearson: E-RG: overall speed: Rho = 0.3728, p = 0.0664, E-RG basal speed: Rho = 0.3559, p = 0.0808, E-RG apical speed: Rho = 0.3638, p = 0.0738, M-RG: overall speed: Rho = 0.4484, p = 0.1078, M-RG basal speed: Rho = 0.2774, p = 0.3370, M-RG apical speed: Rho = 0.5438, p = 0.0444. Linear fit: dashed line for apical, solid for basal motion. 25 E-RG rosettes and 14 M-RG rosettes were analyzed. (TIF) [file pcbi.1004453.s011.tif]

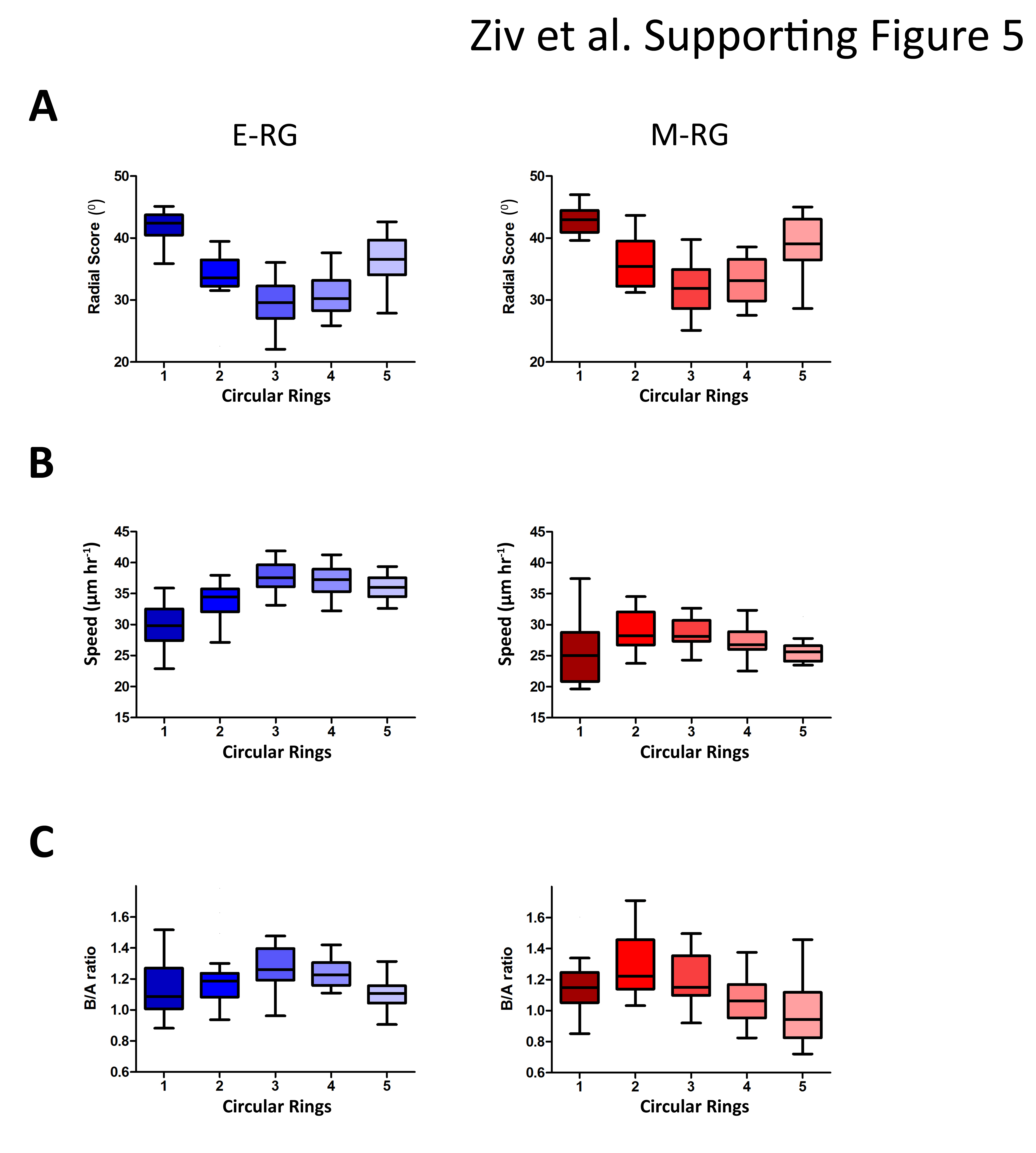

Supplement: S5 Fig — 5 circular rings with equal widths are defined from each rosette center. Ring #1 is closest to the rosette center while #5 to the periphery. E-RG (left) versus (M-RG) for RS (A), Speed (B) and B/A (C). 25 E-RG rosettes and 14 M-RG rosettes were analyzed. (TIF) [file pcbi.1004453.s012.tif]

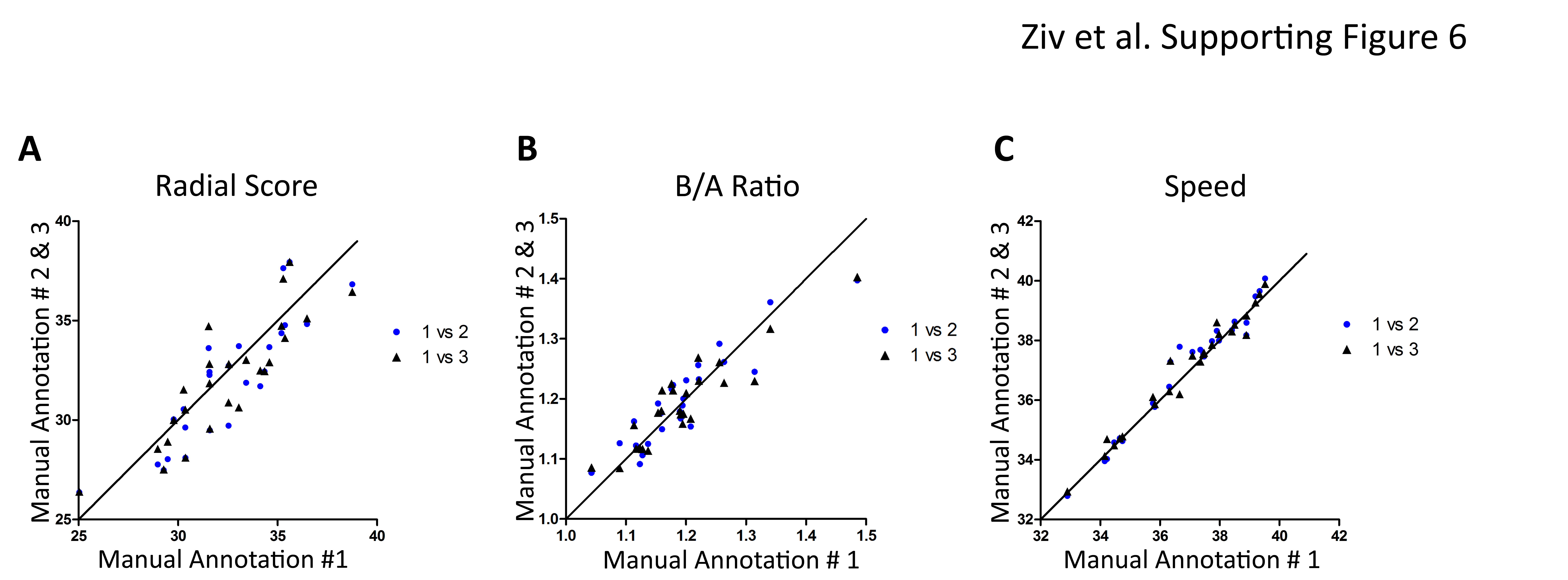

Supplement: S6 Fig — Assessment of the variability in kinetic measures caused by different manual rosette annotations. Three independent manual annotations of rosette centers and contours were used to calculate RS (A), B/A ratio (B) and speed (C). In each panel, x-axis represents the measure of the first annotation that was used to calculate the measures reported in Figs 3–7, y-axis displays the measures of the two additional annotations. The black line Y = X is used as guideline. Pearson correlation was used for statistics comparing 3 pairs of annotations for each measure. A. RS: Rho = 0.8773, p = 8.6006e-09; Rho = 0.8605, p = 3.4538e-08; Rho = 0.9610, p = 2.4466e-14. B. B/A ratio: Rho = 0.9151, p = 1.5085e-10; Rho = 0.9147, p = 1.5845e-10; Rho = 0.9531, p = 1.9627e-13. C. Speed: Rho = 0.9812, p = 6.0661e-18; Rho = 0.9844, p = 7.4069e-19; Rho = 0.9807, p = 8.4915e-18. (TIF) [file pcbi.1004453.s013.tif]
